# Synthesis, cytotoxic screening and molecular docking, DFT calculation of novel furan-heterocyclic derivatives as insecticidal agents

**DOI:** 10.1038/s41598-025-06248-7

**Published:** 2025-06-27

**Authors:** Hager G. El-kasabi, Margret M. Girges, Samira A. Abd El-Salam, Ahmed E. Suliman, Ghada E. Abdel-Ghani

**Affiliations:** 1 Department of Chemistry, Faculty of Science, Mansoura University, Mansoura, Egypt; 2 Plant Protection Research Institute, Agricultural Research Center, Giza, 12618 Egypt; 3 Burg Al-Arab Petroleum Company (Burapetco, 204 A ST 287, New Maadi, Cairo Egypt

**Keywords:** Furan-2-carbaldehyde thiosemicarbazone, Insecticidal activity, DFT, Molecular docking, Biochemistry, Biological techniques, Biotechnology, Cancer

## Abstract

The insecticidal furan-2-carbaldehyde thiosemicarbazone **(1)** as staring compound underwent a nucleophilic substitution reaction with different reagents, chloroacetyl chloride, chloroacetic acid. 1,4-dibromobutane-2,3-dione and also, with different activated reagents 2-cyanoacetohydrazide, phthalic anhydride, and 2-chloroquinoline-3-carbaldehyde as good yields. The structures of these compounds were confirmed by elemental and spectral analyses. The majority of the synthesized compounds were assessed for their insecticidal activity towards three insects, *Cryptoblabes gnidiella*, *Retithrips syriacus* and* Spodoptera frugiperda* under laboratory conditions and promising results were obtained, with encouraging outcomes observed. Compounds **5, 7, 9, 11** and **15** were found to the most effective than other compounds on all insects. Also, *R. syriacus* insects are more affected than *C. gnidiella* and *S. frugiperda* after one day of treatment with LC_50_ values 15.68, 18.90, 58.04, 17.81, and 42.21 μg/mL respectively, comparing with positive control LC_50_, 8.90 μg/mL. Furthermore, biochemical parameters of five enzymes of *S. frugiperda*; Acid Phosphatase, alkaline phosphatase, aspartate transferase, alanine transaminase, and acetylcholinesterase enzymes were conducted at LC_50_ value of the highly toxic compounds. Density functional theory calculations were employed to optimize the molecular geometry and compute the electrostatic potential, complemented by molecular docking to predict the most acceptable score and root mean square deviation and affinities of the synthesized compounds.

## Introduction

**Fig. 1:**
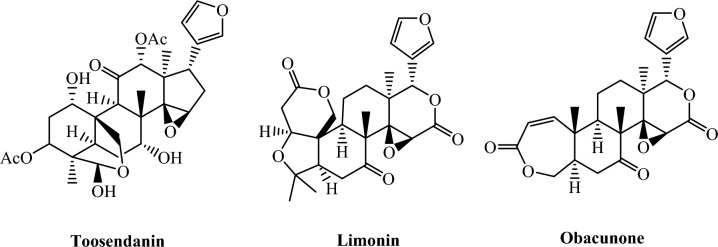
Furan-containing natural products: toosendanin, limonin and obacunone as potent insecticidal agents.

Furan derivatives are a main class of heterocyclic compounds that have important biological properties and present in pharmaceutical products^1^. These compounds have many activities such as anticancer^2^, antimalarial^3^, and antioxidant activities^4^, and also showed insecticidal activities such as the molt inhibiting activity^5^ and the antifeedant activity^6^. Furan ring is widely present in many active natural products^7^ such as toosendanin (naturally extracted insecticidal agents)^8^ and limonin^9^ (Fig. 1). Obacunone is another natural limonoid with a furan ring from many species of plants^10^, such as Citrus and Dictamnus angustifolius. In agricultural and public health contexts, controlling insect populations is crucial to preventing the spread of diseases and protecting crops^11^. Grapes are among the most prized traditional fruit kinds, dating back to ancient times. The unique natural products that grapes offer are becoming more and more helpful every day. They may be used to make a variety of industrial products as well as vital medications to treat a variety of diseases. The grape crop is affected by many insects such as black vine thrips, *Retithrips syriacus* Mayet and Honey dew moth, *Cryptoblabes gnidiella* Miller. *R. syriacus* is a polyphagous thrips that feeds on grapevine, avocado, apple, walnut, and pear in several countries^12^. Nymphs and adults thrips led to grey color on the leaves, accompanied by insects’ excrement that shiny black dots. Leaf wilt and drop are caused by heavy infestations by thrips. Fruits that have been attacked might not grow normally. *Cryptoblabes gnidiella* Mill, an opportunistic honeydew moth (HM), is found in the Mediterranean region^13^. Many hosts, including citrus and vegetable crops, pomegranates, mangoes, grapes, avocados, and more, are attacked by *C. gnidiella*, which has been found all over the world. *C. gnidiella* can harm clusters directly when its larvae eat among the berries and indirectly when it invades injured berries with fungi. Crop output was reduced by 30% as a result of *C. gnidiella.* Around the world, fall armyworms (FAW), *Spodoptera frugiperda*, are a damaging pest to many strategic crops like maize, cabbage, okra and cotton^14^. Originally from North and South America, it has been causing major damage to various cultivated crops, particularly maize harvests, since it began to invade Africa in 2016. FAW may feed on a wide variety of plant hosts, including more than 350 different plant species. It has been estimated that FAW damage causes up to 13 billion US dollars in agricultural losses per year in sub-Saharan Africa^14^. The unique chemical properties of furan and thiazole rings make them promising scaffolds for the development of effective insecticides with enhanced selectivity and potency^15^. Compounds of furan with different heterocyclic units showed activity as insecticidal agents^16^. Thus, controlling these pests and reducing their damage has become necessary and inevitable by using new compounds that reduce the resistance of these pests to traditional pesticides. Therefore, the current study was planned to synthesize some new heterocyclic compounds containing a furan moiety and screening their insecticidal activity against *R. syriacus*, *C. gnidiella* and* S. frugiperda* and evaluate some biochemical parameters by using the powerful of computational approaches to predict the electronic properties of the target molecules were analyzed using DFT and ESP surfaces were generated to identify the regions of electrophilic ad nucleophilic reactivity provided by docking results explained the importance of charge-driven interactions in stabilizing ligand–protein complexes^17^.

## Results and discussion

### Chemistry

**Fig. 2:**
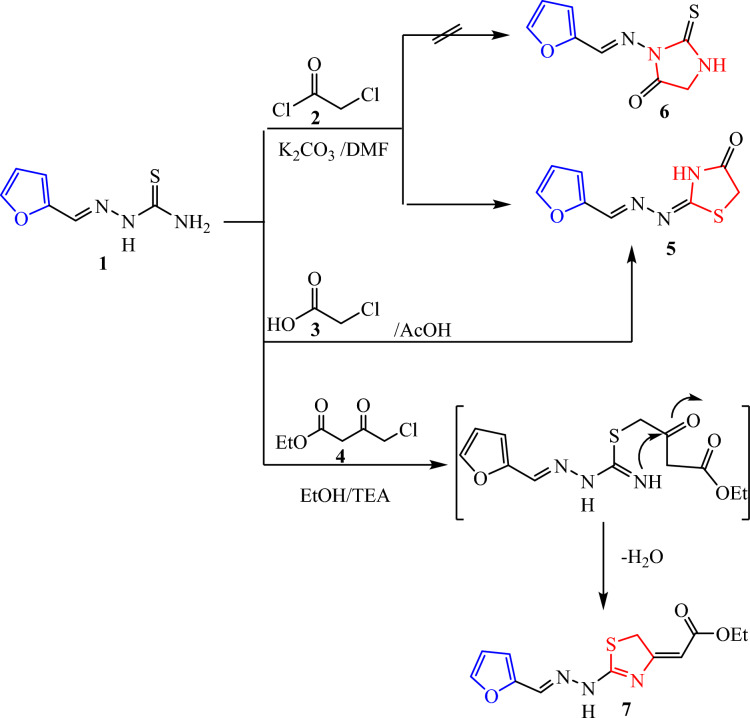
Synthesis of thiazole derivatives of compound **5** and **7**.

**Fig. 3:**
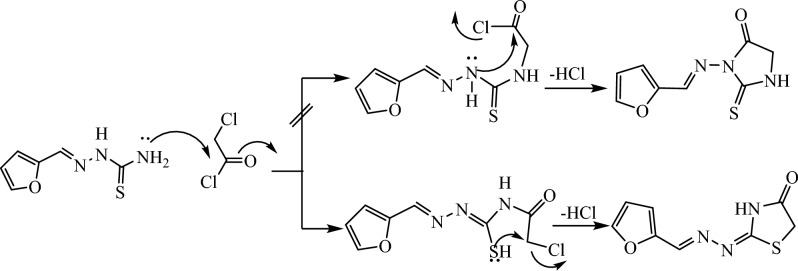
A plausible mechanism for the synthesis of compounds **5**.

**Fig. 4:**
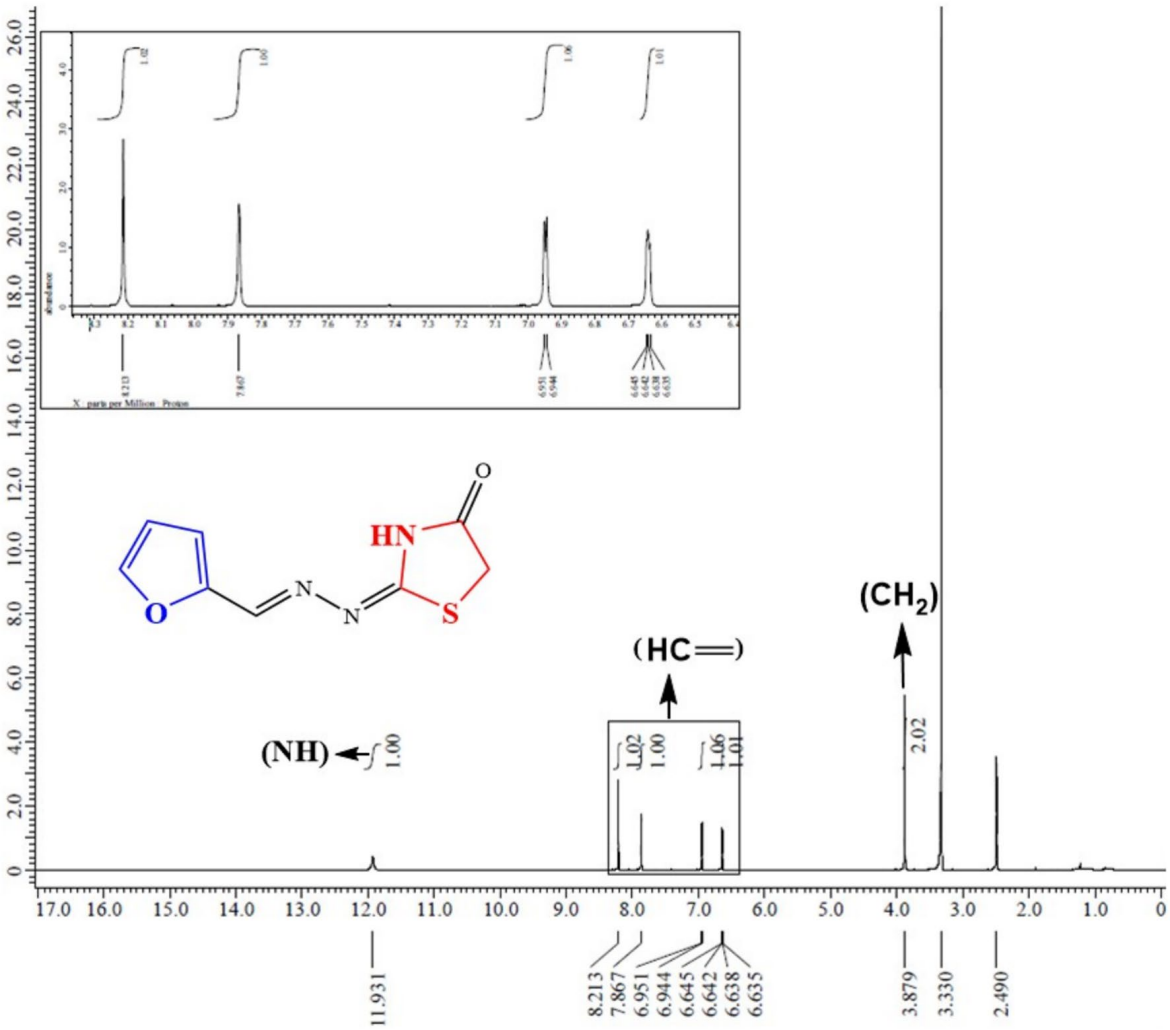
The ^1^H-NMR explanation of compounds **5**.

The key intermediate furan-thiosemicarbazone **1** has a broad range of significant biological activities including insecticidal activity^18^ which was prepared by the furan-2-carbaldhyde with thiosemcarbazide in ethyl alcohol and acetic acid^19^ and characterized by mass spectrometry and FT-IR and NMR spectroscopic techniques. Thiosemicarbazone **1** chemical reactivity with α-halo-compounds was examined in order to create a number of novel thiazole systems (Fig. 2). Thus, treatment of thiosemicarbazone derivative **1** with some carbon electrophiles like chloroacetyl chloride in stirring with dimethylformamide and potassium carbonate through elimination of thiol and cyclization to thiazolidin-4-one not 2-thioxoimidazolidin-4-one through the formation of intermediate (Fig. 3) which confirmed with elemental and spectral data agree with the expected thiazolidin-4-one **5** structures. In another way, we can prepare thiazolidin-4-one* 5* by refluxing thiosemicarbazone **1** and chloroacetic acid in glacial acetic acid for 10 h (Fig. 2). The spectra and elemental analysis of compound **5** were in accordance with suggested structure. The IR spectrum of compound **5** showed stretching bands for NH and CO groups at ν 3435 and 1712 cm^−1^, respectively. The ^1^H NMR spectra of **5** showed new singlet signal at δ 3.88 ppm of methylene protons and showed signals at δ 8.21 and 11.93 ppm for the olefinic CH=proton and NH (Fig. 4). Its ^13^C NMR spectra revealed signal at δ 33.04, 149.28 and 174.02 ppm corresponding to CH_2,_ (N=CH) and CO groups. The mass of compound **5** was determined by mass spectrometry is equal to the calculated value m/z = 209.03 (M^+^, 10.02%). In a similar manner, when thiosemicarbazone** 1** was allowed to react with the keto-ester as (4-chloro-ethyl acetoacetate) which the former attack led to the elimination of hydrogen chloride molecule, while the later led to the removal of water molecule. Thus, conduction of the keto-ester **4** with thiosemicarbazone **1** in boiling ethanol including triethylamine in it afforded in each case a single isolable product, namely, ethyl-2-(2-(2-(furan-2-ylmethylene)hydrazinyl) thiazol-4(5*H*)-ylidene) acetate **7** (Fig. 2). The ^1^H NMR spectrum of **7**, as a representative example of the prepared thiazole acetate, revealed three singlet signals due to acetate (CH_3_) and two CH_2_ protons at δ 1.20, 3.58 and 4.08 ppm, respectively, and three (H-furan) protons at δ 6.59–7.79 ppm, in addition to three singlet signals due to methine (=CH), and NH protons at δ 7.87 and 11.97 ppm, respectively. Its ^13^C NMR spectra revealed signal at δ 14.45, 14.58 and 60.74 ppm corresponding to CH_3_ and two CH_2_ groups. The mass spectrum of compound **7** showed the molecular ion peak at m/z = 279 corresponding to the molecular formula C_12_N_3_H_13_O_3_S.

**Fig. 5:**
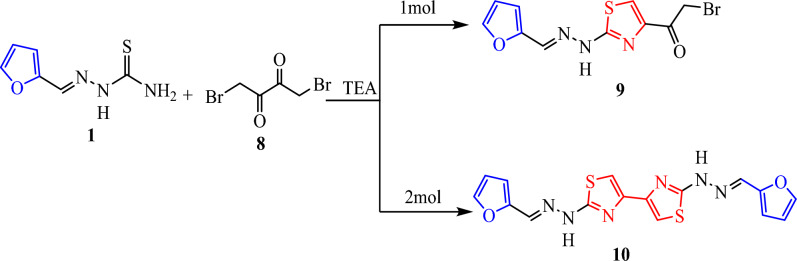
Synthesis of thiazole **9** and bis-thiazole **10**.

**Fig. 6:**
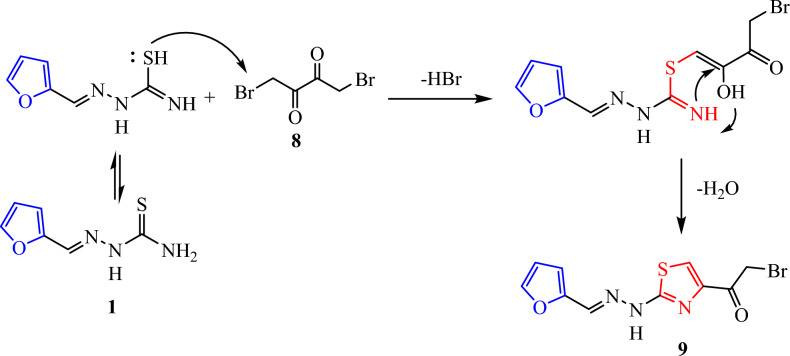
A plausible mechanism for the synthesis of compounds **9**.

On the other hand, we commenced our study on the reactions of one mole (1:1) thiosemicarbazone **1** with 1,4-dibromobutane-2,3-dione **8** in refluxing ethanol (6–10 h) in the presence of a basic catalyst such as TEA furnish the respective thiazole derivatives **9**. While, the two moles (2:1) of thiosemicarbazone **1** with 1,4-dibromobutane-2,3-dione **8** give 2,2′-bis(2-(furan-2-ylmethylene) hydrazinyl)-4,4′-bithiazole **10** (Fig. 5)**.** The plausible mechanism for the synthesis of compounds **9 in** (Fig. 6). Structure **9** and **10** were elucidated via elemental analysis and spectral data. The IR spectra of **9** ascertained their structures by the appearance of the characteristic bands at ν 3405 for NH, and 1624 cm^−1^ due to C=O. Its ^1^H NMR spectrum showed new singlet signals at δ 3.76, 7.98 and 12.21 due to methylene group, (thiazole -H5) and NH group, respectively. While, the IR spectra of **10** showed absence of band of C=O at ν 1624 cm^−1^ and its ^1^H NMR spectrum confirmed the structures of bis- thiazole 1**0** by a peak for two methine (=CH) of (thiazole -H5) and NH protons at δ 6.98 and 12.15 ppm, respectively. The mass spectrum of compound **10** showed the molecular ion peak at m/z = 382.26 (M^+^, 56.09%), corresponding to the molecular formula C_16_H_12_N_6_O_2_S_2_.

**Fig. 7:**
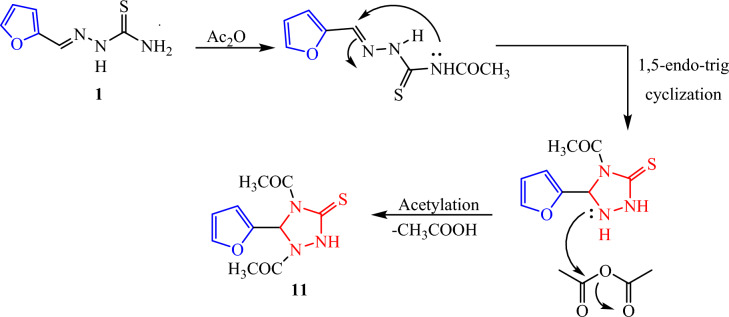
Synthesis of 3-thioxo-1,2,4-triazolidine **11**.

Medicinal chemists have been interested in 1, 2, 4-triazole-based derivatives for the past ten years due to their intriguing pharmacophoric characteristics^20^. A wide range of biological activities, such as antibacterial^21^, antiviral^22^ anticancer,^23^ antioxidants,^24^ etc., are demonstrated by the 1,2,4-triazoles’ electron-rich nature, which aids in their binding with different biological targets and enzymes. Over time, the use of 1,2,4-triazoles in the creation of new molecules has grown significantly. There are several triazole-containing medications on the market with a variety of uses. Whereas, hetero-cyclization of thiosemicarbazone **1** in the presence of acetic anhydride without catalyst resulted thiosemicarbazone intermediates were cyclized into 1,3,4- thiadiazolines **11**. A plausible mechanism for the reaction of compound **1** with acetic anhydride is shown in (Fig. 7)^25^. The IR spectrum of **11** displayed absorption bands at ν 3213 1708, 1694 and 1288 cm^−1^ for NH, two C=O, and C=S groups. Moreover, it’s ^1^H NMR spectrum showed new singlet signals for two CH_3_, CH-triazole and NH protons at δ 2.04, 2.14, 6.90 and 11.79 ppm, respectively (Fig. 7). The mass spectrum of compound **11** afforded the molecular ion peak at m/z = 253 corresponding to the molecular formula C_10_H_11_N_3_O_3_S.

**Fig. 8:**
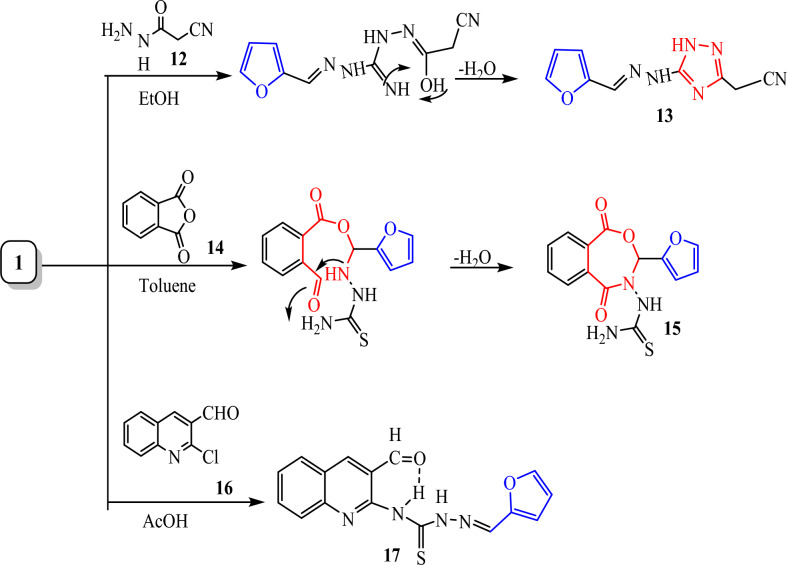
Synthesis of furan derivatives with different moieties of compound **13**, **15** and **17**.

**Fig. 9:**
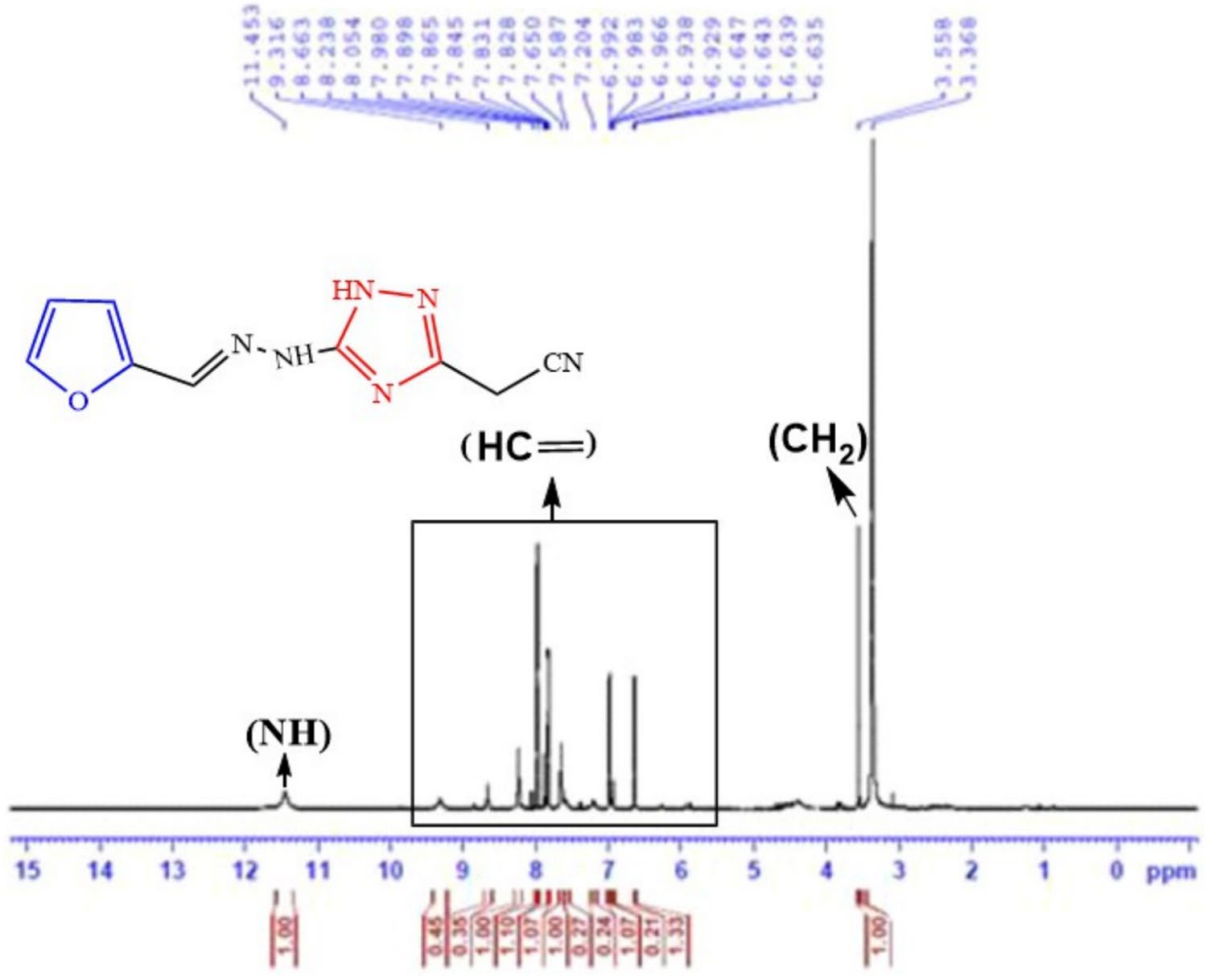
The ^1^H-NMR explanation of compounds **13**.

Also, the 1,2,4-triazolidine ring was synthesized by reaction of thiosemicarbazone **1**, 2-cyanoacetohydrazide in ethanol in the presence of trimethylamine under reflux led to formation of hydrazinyl 1,2,4-triazol-3-yl) acetonitrile **13** in a good yield as brown crystals (Fig. 8). Spectroscopically, the IR spectrum of **13** lacked the carbonyl absorption but disclosed absorption bands for two NH, and new CN groups at ν = 3367 and 2260 cm^−1^, respectively. Further, it’s ^1^H NMR spectrum offered two exchangeable singlet signals for CH_2_ and two NH protons at δ 3.56, 9.31 and 14.45 ppm, respectively (Fig. 9). The mass spectrum of compound **13** showed the molecular ion peak at m/z = 216.20 corresponding to the molecular formula C_9_H_8_N_6_O.

Cyclization of furan-2-carbaldehyde thiosemicarbazone **(1)** depended on the cyclizing agent and the reaction conditions. Thus, thiosemicarbazone derivative **1**, which underwent ring closure with phthalic anhydride and 2-chloroquinoline-3-carbaldehyde in different solvents yielded dihydrobenzo [e][1,3] oxazepine-4(3*H*)-carbothioamide derivatives** 15** and* N*-(3-formylquinolin-2-yl)-2-(furan-2-ylmethylene) hydrazine-1-carbothioamide **17** showed in (Fig. 8). The analytical and spectral data of compound **15** and **17** were consistent with the proposed structure. The IR spectrum of **15** and** 17** showed the presence of a new band at ν 1689 and 1751 cm^−1^ attributed to a carbonyl group of oxazepine-4(3*H*)-carbothioamide **15** and 3-formylquinoline **17** respectively. The ^1^H NMR spectrum of compound **15** showed no evidence of thiazolo-methylene protons and showed a multiple signal at δ 7.25–7.98 and 8.24 ppm for the aromatic protons and methine proton due to protons oxazepine. The ^13^C NMR spectra of **15** showed signals at δ 61.61, 145, 149, 169 and 178 ppm corresponding to CH, C=CH, N=CH and C=O groups, respectively. The ^1^H NMR spectrum of **17** showed a new signal at δ 9.09 ppm attributed to the CHO proton and two secondary NH at δ 12.4 and 14.17 ppm. The mass spectrum of compound **15** showed the molecular ion peak at m/z = 317.32 corresponding to the molecular formula C_14_H_11_N_3_O_4_S. The mass spectrum of compound **17** showed the molecular ion peak at m/z = 325.26 corresponding to the molecular formula C_16_ H_12_ N_4_SO_2._

### Biological screening (cytotoxic activity)

#### Insecticidal activity

**Fig. 10:**
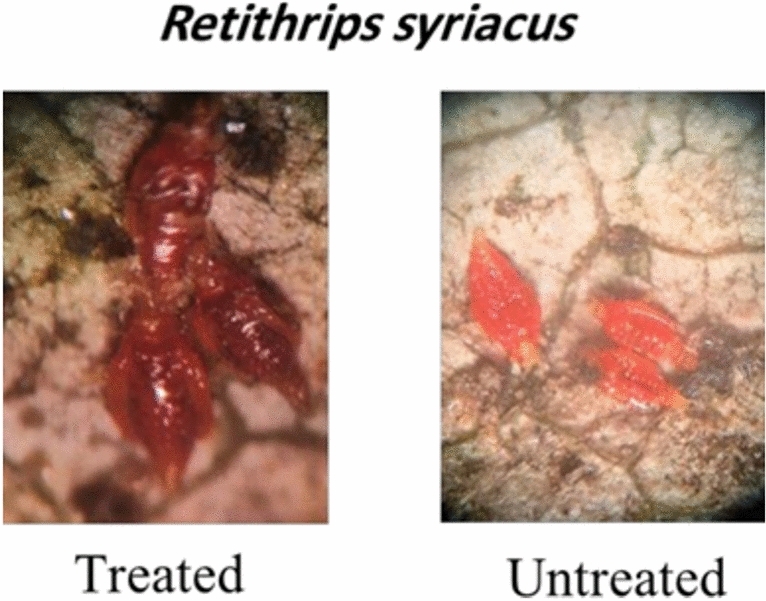
Result of high active furan compounds **5, 7, 9, 11** and **15** against *R. syriacus* insect.

**Fig. 11:**
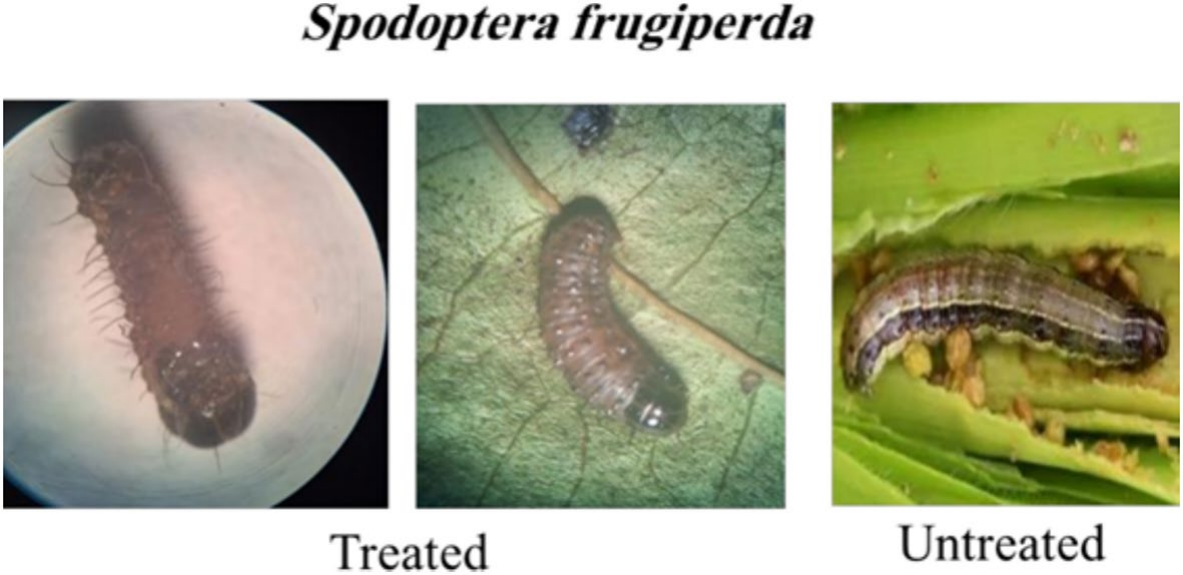
Result of high active furan compounds **5, 7, 9 and 11** against *S. frugiperda.*

The toxicity of the new furan derivatives was evaluated against Retithrips syriacus, Cryptoblabes gnidiella, and Spodoptera frugiperda under laboratory conditions. A comparison between all compounds and the positive control (Acetamiprid 20% SP and Indoxacarb) was done by toxicity index. All compounds showed activity against all insects, especially toward R. syriacus (Figs. 10, 11) (Tables 1, 2 and 3). The results showed that compounds **5, 7, 9, 11** and **15** were more effective than other compounds with LC50 values 15.68, 18.90, 58.04, 17.81, and 42.21 μg/mL, respectively, compared with positive control LC50, 8.90 μg/mL against R. syriacus after 24 h from treatment, and for C. gnidiella with LC_50_ values 35.67, 51.99, 37.85, 56.46, and 106.82 μg/mL respectively, comparing with Indoxacarb LC_50_, 13.29 μg/mL after 3 days of treatment. Only compounds **5, 7, 9,** and **11** were the most toxic toward S. frugiperda, with LC_50_ values 37.75, 58.04, 48.17, and 53.51 μg/mL respectively, comparing with positive control Indoxacarb LC_50_, 15.85 μg/mL after 3 days of treatment.

**Table 1:** Toxicity of furan derivatives on Grape Thrips (*Retithrips syriacus*) after 24 h under laboratory conditions.

Treatments	LC50 (μg/mL)	confidence intervals 95% (μg/mL)		LC90 (μg/mL)	confidence intervals 95% (μg/mL)		Slope ± S.E	r-values	Toxicity Index (%) at LC50 value
		Lower	Upper		Lower	Upper			
1	96.73	72.39	128.72	463.98	299.92	987.03	1.88 ± 0.29	0.9763	9.20
5	15.68	11.75	20.40	67.41	46.01	127.38	2.02 ± 0.31	0.9934	56.76
7	18.90	14.10	25.15	91.44	59..09	194.86	1.87 ± 0.29	0.9687	47.09
9	58.04	43.44	77.24	278.40	179.96	592.24	1.88 ± 0.29	0.9785	15.33
10	211.05	156.48	286.79	1111.65	687.75	2622.62	1.78 ± 0.29	0.9447	4.22
11	17.81	12.74	24.40	106.29	64.22	269.35	1.65 ± 0.28	0.9274	49.97
13	173.29	125.80	233.7	940.35	589.87	2165.72	1.74 ± 0.29	0.9875	5.14
15	42.21	31.30	57.36	222.33	137.55	524.52	1.78 ± 0.29	0.9787	21.09
17	184.18	132.86	262.17	1194.81	672.46	3601.47	1.58 ± 0.28	0.9914	4.83
Acetamiprid 20%SP	8.90	6.62	11.80	42.74	27.86	89.37	1.88 ± 0.29	0.9897	100

**Table 2:** Toxicity of furan derivatives on Honey dew moth (*Cryptoblabes gnidiella*) after 3 days under laboratory conditions.

Treatments	LC50 (μg/mL)	Confidence intervals 95% (μg/mL)		LC90 (μg/mL)	Confidence intervals 95% (μg/mL)		Slope ± S.E	r-values	Toxicity Index (%) at LC50 value
		Lower	Upper		Lower	Upper			
1	217.17	160.35	297.77	1187.90	721.78	2929.11	1.74 ± 0.29	0.9900	6.12
5	35.67	26.14	47.91	186.70	118.20	419.43	1.78 ± 0.29	0.9845	37.26
7	51.99	37.74	70.11	282.11	176.96	649.73	1.74 ± 0.29	0.9854	25.56
9	37.85	27.48	51.70	216.06	131.64	533.28	1.69 ± 0.28	0.9955	35.11
10	252.07	204.26	304.81	754.74	551.87	1385.15	2.69 ± 0.49	0.9475	5.27
11	56.46	40.30	78.23	353.43	208.17	955.06	1.61 ± 0.28	0.9789	23.54
13	254.56	202.23	314.27	857.54	595.92	1829.31	2.43 ± 0.47	0.9667	5.21
15	106.82	79.44	141.62	512.79	334.34	1072.38	1.88 ± 0.29	0.9959	12.44
17	307.52	250.21	371.60	911.15	667.14	1667.40	2.72 ± 0.49	0.9544	4.32
Indoxacarb	13.29	9.82	17.60	65.16	42.07	142.54	1.86 ± 0.30	0.9781	100

**Table 3:** Toxicity of furan derivatives on Fall Armyworm (*Spodoptera frugiperda*) after 3 days under laboratory conditions.

Treatments	LC50 (μg/mL)	Confidence intervals 95% (μg/mL)		LC90 (μg/mL)	Confidence intervals 95% (μg/mL)		Slope ± S.E	r-values	Toxicity Index (%) at LC50 value
		Lower	Upper		Lower	Upper			
1	250.46	186.59	347.39	1340.65	804.55	3390.65	1.76 ± 0.29	0.9956	6.33
5	37.75	28.53	49.64	168.53	111.87	337.20	1.97 ± 0.30	0.9861	41.99
7	58.04	43..94	76.41	259.42	171.71	522.03	1.97 ± 0.30	0.9938	27.31
9	48.17	35.59	63.51	227.19	150.48	459.73	1.90 ± 0.30	0.9908	32.90
10	399.25	288.73	551.91	2395.81	1422.15	6311.01	1.65 ± 0.28	0.9787	3.97
11	53.51	39.21	71.87	280.05	177.30	629.16	1.78 ± 0.29	0.9860	29.62
13	367.73	276.01	485.25	1695.39	1116.52	3458.03	1.93 ± 0.30	0.9780	4.31
15	388.18	291.79	514.60	1814.34	1182.79	3782.48	1.91 ± 0.30	0.9777	4.08
17	398.68	298.43	532.37	1933.47	1240.88	7179.58	1.87 ± 0.29	0.9800	3.98
Indoxacarb	15.85	10.58	23.28	36.68	28.10	52.76	2.48 ± 0.35	0.9798	100

Heterocyclic are considered the key of agrochemicals design due to their role in propesticidal activity, their capacity to spread pharmacophoric functions in 3D space, and their potential as a bioisosteric substitute for functional groups or other rings^26^. Furan derivatives are a main class of heterocyclic compounds that have important insecticidal propertien^5^. The nature and position of the substituents of thiazole derivatives are important to analysis its effects as insecticidal properties. the presence of electron-withdrawing groups such as bromine, acetate and acetyl groups were found to enhance the pesticides activity^27^.

#### Effect of some compounds on *S. frugiperda* enzymes activity

The effect of the highly toxic compounds **5, 7, 9** and** 11** on the activity of *S. frugiperda* enzymes (AST, ALT, ALP, ACP, and AChE) (Fig. 11) was measured as shown in (Table 4). There was no significant difference in all treatments of the AST enzyme except compound **7,** which exhibited a strong significant decrease compared with the control with values of 22.0 and 221.0 U/L, respectively. Compounds **9** and** 11** showed a strong significant decrease for ALT enzyme compared with the control with values of 91.7, 122.7 and 227.0 U/L, respectively. The results of the acid phosphatase enzyme showed a significant increase with all tested compounds. While ALP activity slightly increased in all treatments. AChE enzyme activity showed highly significant activation with all tested compounds, especially compounds **7**, **9** and **11** (Table 4).

**Table 4:** Effect of compounds 5, 7, 9 and 11 on some biochemical parameters of *S. frugiperda*.

Treatment	AST (U/L) ± SE	ALT (U/L) ± SE	ALP(U/L) ± SE	ACP (U/L) ± SE	AChE (U/L/min) ± SE
Control	221.0 ± 5.29^a^	227.0 ± 2.65^a^	11.24 ± 0.21^c^	2.77 ± 0.15^c^	320.0 ± 2.31^e^
5	211.7 ± 2.73^a^	219.0 ± 3.06^ab^	13.72 ± 0.17^b^	6.14 ± 0.13^a^	1494.0 ± 27.87^a^
7	22.0 ± 4.04^b^	210.3 ± 6.94^b^	13.49 ± 0.24^b^	4.05 ± 0.22^b^	1376.3 ± 18.35^b^
9	212.3 ± 3.53^a^	91.7 ± 5.21^d^	15.82 ± 0.26^a^	6.05 ± 0.12^a^	392.7 ± 14.99^d^
11	213.3 ± 5.17^a^	122.7 ± 4.10^c^	11.27 ± 0.17^c^	3.67 ± 0.09^b^	1278.3 ± 32.10^c^
LSD (0.05)	13.44	14.67	0.67	0.46	68.66

LSD: least significant difference; SE: standard error; a, b, c, d, and e: Duncan’s letters. The same letter in the same column means non-significant.

Since the transmission enzymes AST and ALT are important for the Krebs cycle as well as the transfer of proteins and amino acids, any alteration in their activity indicates that the rate of respiration and oxygen consumption were impacted by^28^. The detoxifying enzymes (ACP, ALP, and AChE) were found to react against tested compounds that exhibit insecticidal activities^29^. ACP enzyme is especially crucial for the cytolysis of tissues in insects during development. Also, any changes in the development of both phosphatase enzymes are mirrored in changes in the amount of acid-soluble phosphorus^30^. In order to stop nerve impulses, AChE can catalyse the hydrolysis of the neurotransmitter acetylcholine in the neurological systems of different organisms^31^. Resistance in lepidopteran pests appears to be primarily conferred by increased activity of the detoxifying enzyme AchE.

### Computational methods

#### Quantum calculation analysis

**Fig. 12:**
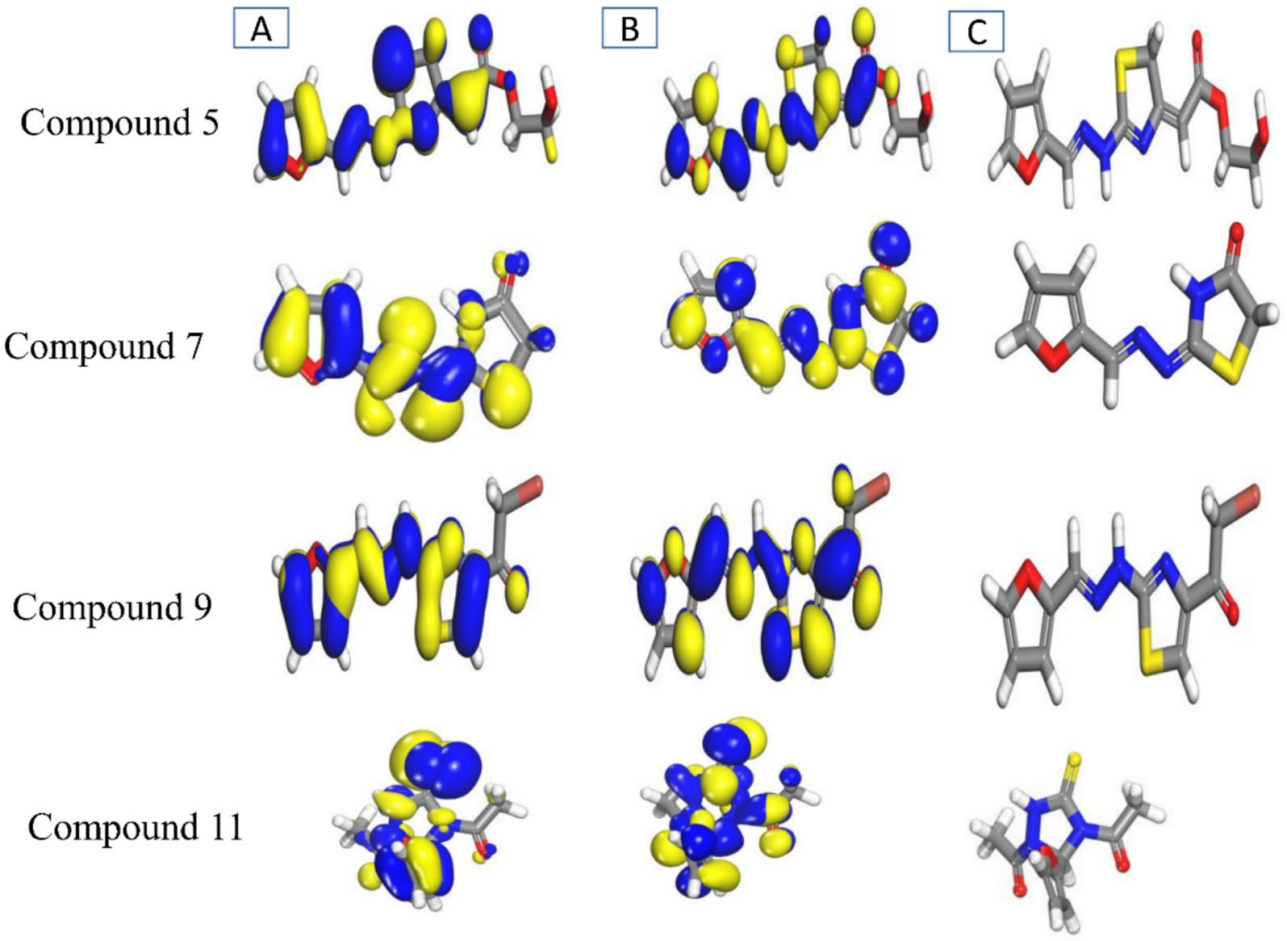
The density distributions of HOMO (**A**), LUMO (**B**) and prepared compound (**C**) outline the electronic distributions of DFT parameters.

In this study, DFT calculations were performed using Dmol^3^ module to investigate the electronic characters and reactivity of synthesized molecules, with considering the possibility of insecticidal activity. The power of DFT lie in its ability to describe the electron correlation effects while maintaining computational powerful. The function GGA with PBE was Implemented to meet with double numerical plus polarization (DNP) basis set, to optimize molecular geometry and compute frontier molecular orbitals (FMOs)^32^. The values of HOMO and LUMO was implemented to identify the reactive sites, with the energy gap (ΔE = E_LUMO_ − E_HOMO_) to indicate the chemical reactivity^33^. The observed energy gap in both gas and liquid phase suggesting high reactivity and charge transfer capability, which are indicator for interaction with biological targets resulting from small value of ΔE gap which facilitates the electron transitions (lower energy barrier), increasing the molecules capacity to donates or accept electrons^34^ (Fig. 12). Demonstrate the prepared compounds **5, 7, 9** and **11** in both gas and liquid phases with density distributions located on over functions groups, π-π bonds and substituted group which enhancing the mentioned energy gap listed in (Table 5) below, however, the value of E_HOMOs_ in indicate the donation of electrons to the nucleophilic behavior that paly a good roll in insecticides specially in neurotransmission system, while the E_LUMOs_ enhancing the electrophilic insecticides react with nucleophilic serine residues in acetylcholinesterase that is important to provide the inhibition process^33^.The absolute electronegativity value can represent as ($\chi$) and absolute hardness element (η) as in following equations^34^.

1 $$\chi = - \mu = \frac{I + E}{2}$$

2 $$\upeta = \frac{{I - {\text{E}}}}{2}$$

**Table 5:** Computed quantum parameters describe the DFT efficiencies.

Compound	EHOMO (eV)	ELUMO (eV)	ΔE (eV)	μ (Debye)	η (eV)	χ (eV)
Gas phase						
**5**	− 5.286	− 3.867	1.419	6.2151	0.7095	4.5765
**7**	− 5.178	− 3.665	1.513	1.2119	0.7565	4.4215
**9**	− 5.333	− 3.754	1.579	7.4991	0.7895	4.5435
**11**	− 5.211	− 3.43	1.781	2.4983	0.8905	4.3205
Liquid phase						
**5**	− 5.452	− 3.5	1.952	11.6393	0.976	4.476
**7**	− 5.334	− 3.816	1.518	1.2684	0.759	4.575
**9**	− 5.303	− 4.046	1.257	10.4064	0.6285	4.6745
**11**	− 5.535	− 3.594	1.941	3.7428	0.9705	4.5645

The electronic properties of evaluated compounds **5, 7, 9** and **11** in gas and liquid phases focusing on their HOMO–LUMO energy gaps (ΔE), dipole moments (μ), and reactivity descriptors (η, χ)^35^,^36^. The results indicate potential phase-dependent behavior: increasing the dipole moments in liquid phase reveal to solvation effects, while energy gap with compound **9** introduce the smallest ΔE (1.257 eV) and lowest hardness (η = 0.6285 eV) in liquid phase refer to enhancing in electro-transfer potential. The lowest ΔE suggests high chemical reactivity, which has the ability to facilitate interactions with biological targets (e.g., insect enzymes) by active efficient electron donation/acceptance^37^.

**Fig. 13:**
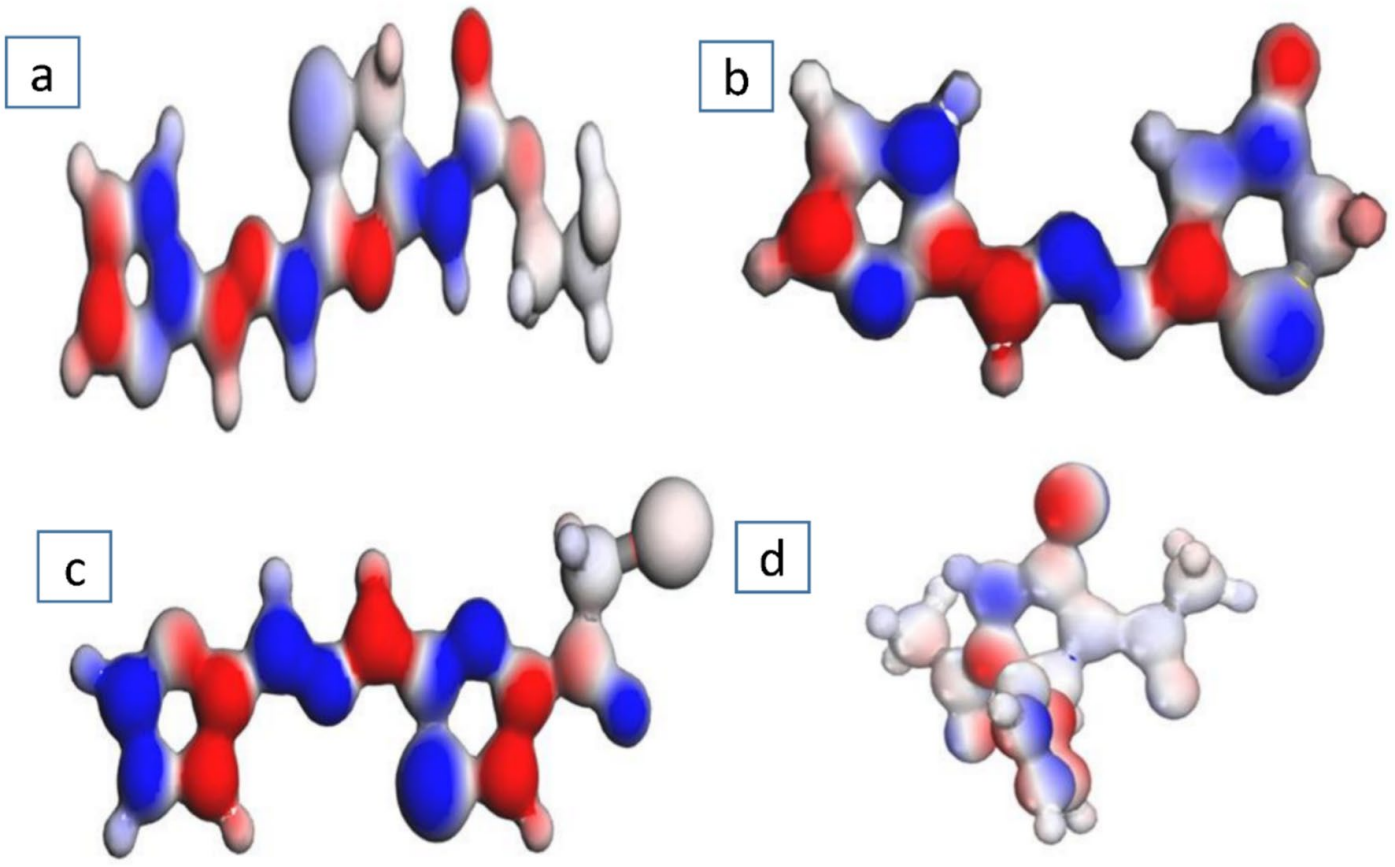
The electrostatic potential (ESP) maps for compounds **5** (**a**), **7** (**b**),** 9** (**c**) and **11** (**d**).

In (Fig. 13), describe the electrostatic potential (ESP) for prepared compounds **5, 7, 9** and **11** this provides the electronic distribution charges across their surfaces. The color maps are indicating to regions of electron-rich (red, negative potential) and deficient in electrons (blue, positive potential) areas and neural zones represented as gray color^38^. This ESP enhancing the result was listed in (Table 5), these insights into electron distribution charges and polarity is important to know how the molecules interact with biological targets.

#### Docking and molecular interaction of synthesized compounds

**Fig. 14:**
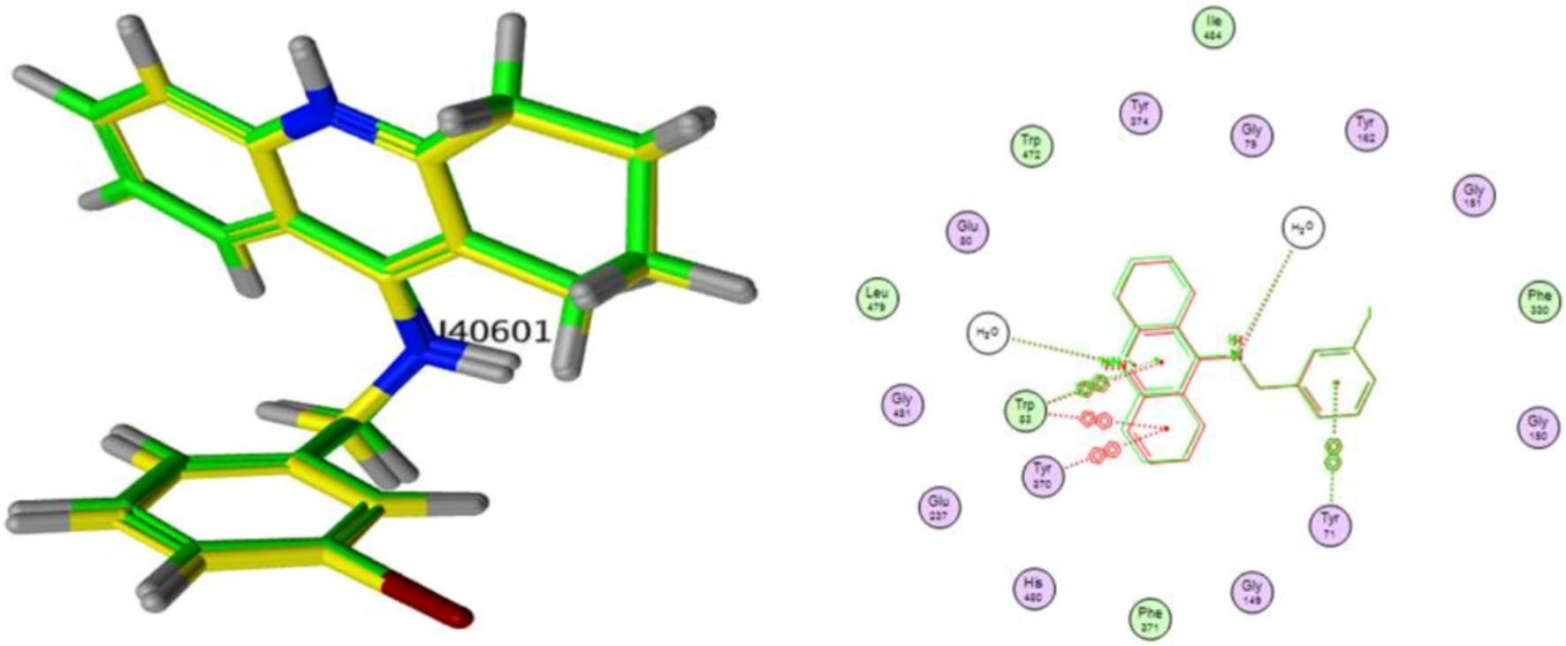
*Left*: superimposition of co-crystal in original protein (green) and docked co-crystal confirmed (yellow), *Right*: original co-crystal with green color overlaying on docked co-crystal with red color.

**Fig. 15:**
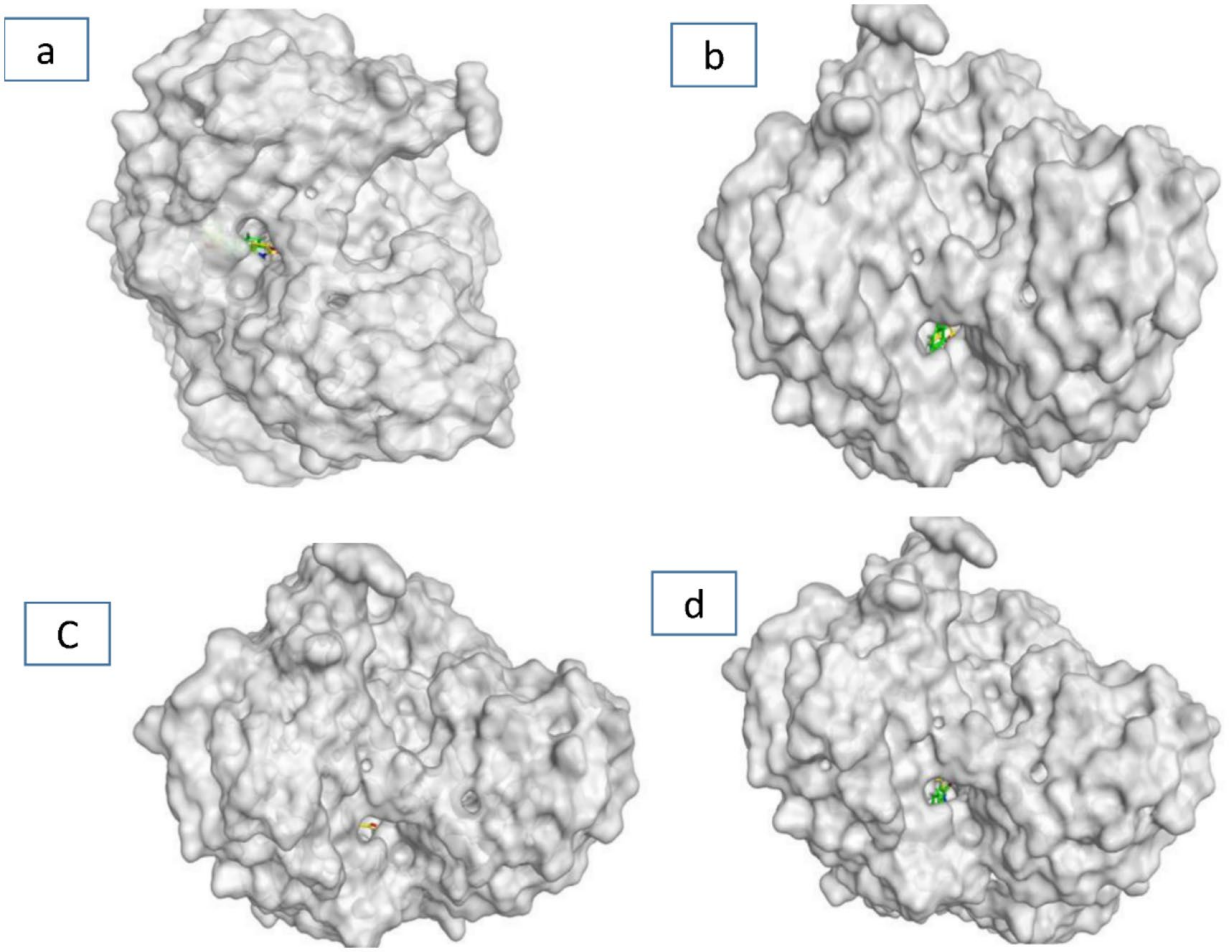
3D protein position for **5** (**a**), **7** (**b**), **9** (**c**) and **11** (**d**) ligands inside the active pocket site of AChE receptors.

**Fig. 16:**
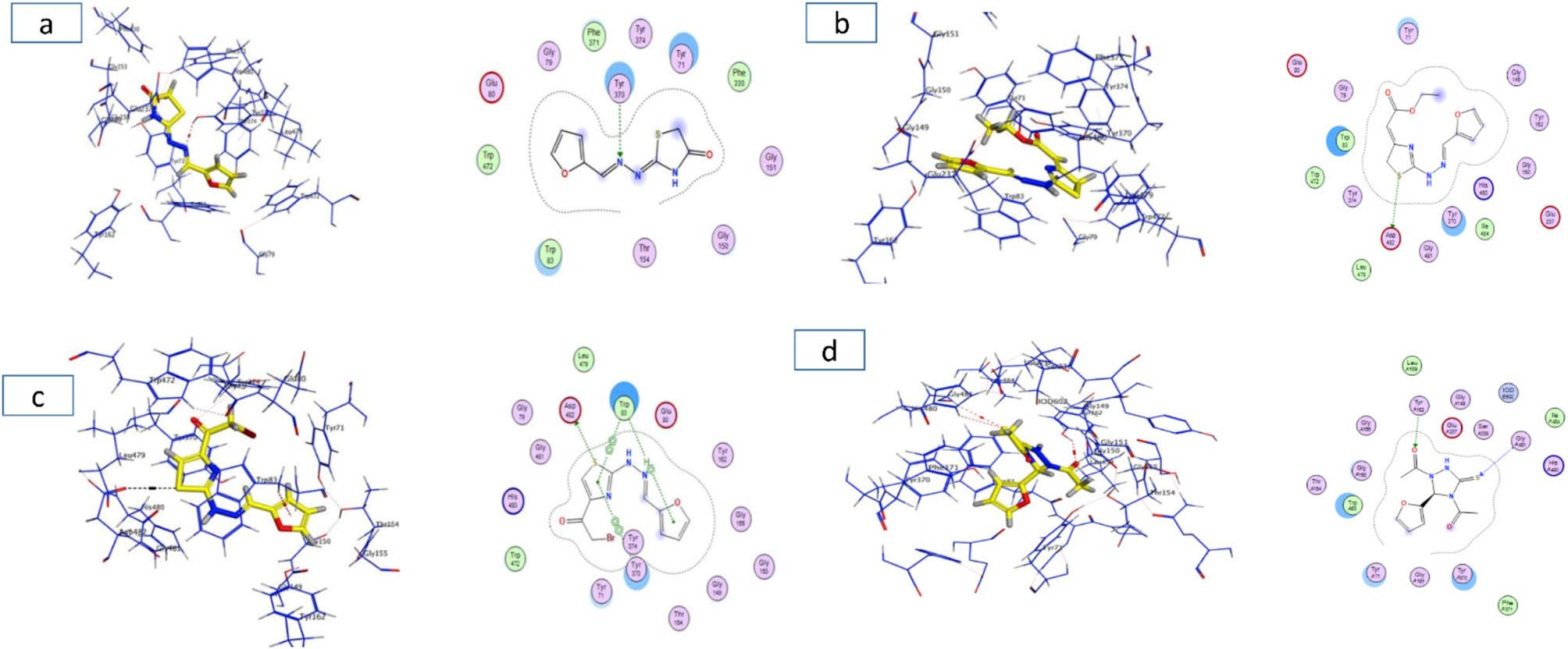
*Left*: 3D structures for **5** (**a**), **7** (**b**), **9** (**c**) and **11** (**d**) interacts with same site of AchE receptors with yellow color, *Right*: 2D structures for **5** (**a**), **7** (**b**), **9** (**c**) and **11** (**d**) the interaction between ligand and receptors.

The synthesized compounds were hypothesized to exert inhibitory effects on *Spodoptera frugiperda* acetylcholinesterase (AChE) represented in the crystal structures of Drosophila melanogaster acetylcholinesterase (Dm AChE)^39^, through that the interactions will target the binding pocket of AChE receptors as showed in (Fig. 14). The molecular docking of acetylcholinesterase (AChE; PDB ID: 6XUY) using MOE software revealed critical insights into ligand-receptor interactions, guided by energy-based scoring and structural parameters. A molecular docking simulation was performed to investigate the interaction of the target compounds **5, 7, 9** and **11** the native ligand within the active site of the spike protein. The simulation confirmed by superimposition of co-crystal and docked co-crystal to insure accurate data as showed in (Fig. 14). Multiple docking poses were generated, exhibiting favorable binding orientations and interactions within the receptor pocket^40^. The poses that demonstrated the most acceptable rmsd_refine (values while maintaining the same binding mode as the native ligand were selected for further analysis. The binding energies and various interactions with the amino acid residues of the spike protein pocket are summarized in (Table 6). The results demonstrated strong binding affinities across tested compound **5, 7, 9** and **11** with S scores ranging from (− 5.42 to − 7.33 kcal/mol) all exceeding the energy cutoff. Notably, ligand **7** exhibited the highest affinity (− 7.33 kcal/mol), forming a hydrogen bond (H-donor) with ASP 482 (distance: 4.30 Å) and π-H interactions with TRP 83. Ligand **9** engaged in diverse interactions, including H-donor bonds with ASP 482 and π-π stacking with TRP 83 and TYR 370, despite weaker energetic contributions (− 0.8 to − 2.4 kcal/mol). The low RMSD values (1.197–2.642 Å) indicated stable ligand conformations within the active site, particularly for **7** (RMSD: 1.510 Å). These findings matching with the biological results indicates that these off-target interactions trigger a rapid physiological response in insects, likely via stress-induced pathway, leading to hyperactivation and overproduction of AchE, The low RMSD values (1.197–1.510 Å) confirm stable ligand docking at these non-canonical sites, suggesting a mechanism where binding to allosteric or peripheral anionic sites (PAS) disrupts feedback regulation^41^, forcing the insect to overexpress AChE or may be unstable interaction between the docked ligand and the target pocket site make the insect resisting the inhibition process by increase the AChE produce as showing in (Figs. 15 and 16).

**Table 6:** The results obtained from docking the prepared compounds with the Co-crystal active site pocket.

Compounds (ligands)	Receptor	S score (kcal/mol)	RMSD (Å)	Bonds between atoms of compounds and residues of active sites of Receptors					
				Atom of compound	Atom of receptor	Involved receptor residues	Type of interaction bond	Distance (Å)	E (kcal/mol)
5	6xuy	− 5.4200	2.642	N 11	OH	TYR 370	H-acceptor	2.99	− 1.5
7		− 7.3300	1.510	S-20	OD1	ASP 482	H-donor	4.30	− 0.6
9		− 6.7954	1.197	S-15	OD1	ASP 482	H-donor	3.61	− 2.4
				5-ring	CB	TRP 83	pi-H	4.01	− 0.8
				5-ring	5-ring	TRP 83	pi-pi	3.55	− 0.0
				5-ring	6-ring	TYR 370	pi-pi	3.88	− 0.0
11		− 6.5467	1.267	S-16	CA	GLY 481	H-accepto r	4.29	− 1.1
				O-18	OH	TYR 162	H-acceptor	2.89	− 2.2

In contrast, the inhibitor **9** exhibited a unique dual-binding profile, interacting strongly with both the co-crystallized inhibitor sites and novel targets, such as ASP 482, through many interaction types, including H-donor bonds (3.61 Å, − 2.4 kcal/mol) and π-π stacking with TRP 83 and TYR 370 (3.55–3.88 Å). This dual-binding mechanism aligns with biological results, where **9** effectively inhibits AChE activity, likely by blocking substrate access to the catalytic triad while simultaneously disrupting allosteric regulation. Finally, we suggest that the insect may try to compensate by producing too much of the enzyme, throwing its metabolism off balance and causing hyperactivity. This dual-action mechanism direct inhibition combined with induced overproduction may be creates a novel pathway for pest control, as the resulting enzymatic overload disrupts normal physiological processes.

## Experimental

### General remarks

Melting points were determined on a Gallenkamp electric apparatus. IR spectra (KBr discs) were measured Thermo Scientific Nicolet iS10 FTIR spectrometer. Also, Bruker’s spectrometer 400 MHz, and JEOL spectrometer 500 MHz and 400 MHz, (^1^H NMR), 125 MHz and 100 MHz (^13^C NMR) were used to estimate NMR spectra in DMSO-*d*_*6*_, as a solvent and an internal standard. Mass spectra were recorded on a GC–MS QP-1000 EX Shimadzu instrument by EI mode at 70 eV. Elemental analyses were determined on PerkinElmer 2400 analyzer.

### Chemistry

#### 2-((-furan-2-ylmethylene)hydrazineylidene)thiazolidin-4-one (5)

**Method I:** To a stirred a solution of Furan-2-carbaldehyde thiosemicarbazone **(1)** (1.69 gm, 0.01mol) in dimethylformamide (5ml), chloroactylchloride (1.59ml, 0.02mol) was added dropwise at room temperature in presence of potassium carbonate (1.382 gm, 0.01mol). Stirring was continued to 10h at (20 °C). then the reaction mixture was poured on cold water. The precipitated solid was collected by filtration and recrystallized from ethanol to give thiazolidin-4-one **5**.

**Method II:** A mixture of Furan-2-carbaldehyde thiosemicarbazone **(1)** (1.69 gm, 0.01 mol) and chloroacetic acid (0.01 mol) in glacial acetic acid (22 ml). The reaction mixture was heated under reflux for 10 h at (150 °C). the precipitated solid filtrated and recrystallized from ethanol/DMF to afford thiazolidin-4-one **5**.

Red powder; yield 70%; mp 210–215 °C; IR (KBr)*ν*/cm^−1^: 3435 (NH), 1712 (C=O), 1644 (C=N). ^1^H NMR (DMSO-*d*_6_): *δ* (ppm): 3.88 (s, 2H, CH_2_), 6.635–6.645 (dd 1H, J = 5 Hz*,* C4-furan), 6.94 (d, 1H, C3-furan), 7.87 (d, 1H, C5-furan), 8.21 (s, 1H, =CH), 11.93 (s, 1H, NH); ^13^C NMR–(δC/ppm): 33.04, 112.33, 115.68, 145.55, 145.86 (2C), 149.28, 174.02; ESI–MS, m/z: 209.48 (M^+^, 10.02%), 133.84 (100%), Anal.Calcd for C_8_H_7_N_3_O_2_S (209.22) Elemental Analysis: C, 45.93; H, 3.37; N, 20.08% found: C, 45.91; H, 4.32; N, 20.05%.

#### Ethyl-2-(2-(2-(furan-2-ylmethylene) hydrazinyl) thiazol-4(5H)-ylidene) acetate (7)

A mixture of thiosemicarbazone **1** (1.69 gm, 0.01 mol), 4-chloro-ethyl acetoacetate (1.36 ml, 0.01 mol) in absolute ethanol (20 ml, 99%) with trimethylamine (3drops) was refluxed for 8 h at (150 °C). After the completion of the reaction had been confirmed by TLC. The reaction was poured onto ice. The precipitated solid was filtered, washed with ethanol and dried. The crude product was recrystallized from ethanol to afford compound **7**. Black powder; yield 80%; mp 95–97 °C; IR (KBr)*ν*/cm^−1^: 3152, 3117 (NH), 1728 (C=O), 1621 (C=N); ^1^H NMR (DMSO-d6): *δ* (ppm): 1.20 (m, 3H, CH_3_), 3.58 (s, 2H, CH_2_), 4.08 (m, 2H, CH_2_), 6.59–6.61 (dd, J = 6.5 Hz, 1H, C4- furan), 6.64 (s, 1H, =CH), 6.78–6.79 (d, 1H, C3-furan), 7.790–7.793 (d, 1H, C5-furan), 7.87 (s, 1H, =CH), 11.97 (s, 1H, NH); ^13^C NMR–(δC/ppm): 14.45, 14.58, 60.74, 106.03, 112.51, 112.73, 131.90, 144.89, 145.33, 149.83, 168.24, 170.50 ; ESI–MS, m/z: 279.45 (M^+^, 13.18%), 95.55 (100%), Anal.Calcd for C_12_H_13_N_3_O_3_S (279.31) Elemental Analysis For C, 51.60; H, 4.69; N, 15.04% found C, 51.61; H, 4.68; N, 15.03%.

#### 2-bromo-1-(2-(2-(furan-2-ylmethylene) hydrazinyl) thiazol-4-yl) ethan-1-one (9)

A mixture of thiosemicarbazone **1** (1.69 gm, 0.01 mol), 1,4-dibromobutane-2,3-dione (2.44gm, 0.01 mol) in absolute ethanol (20 ml, 99%) with trimethylamine (3drops) was refluxed for 7 h at (150 °C) (monitored by TLC), then left to cool. The solid product was filtered off, washed with EtOH and recrystalized from DMF to afford the thiazole derivative **9**. Black powder; yield 78%; mp 180–183 °C; IR (KBr)*ν*/cm^−1^: 3405 (NH), 1624 (C=O), 1564 (C=N); ^1^H NMR (DMSO-d6): *δ* (ppm): 3.76 (s, 2H, CH_2_), 6.61–6.63 (dd, 1H, J = 3 Hz, C4-furan), 6.82–6.83 (d, 1H, C3-furan), 7.82–7.85 (d, 1H, C5-furan), 7.92 (s, 1H, =CH), 7.98 (s, 1H, thiazole), 12.21 (s, 1H, NH); ^13^C NMR–(δC/ppm): 31.19, 104.99, 112.56, 112.84, 144.89, 145.03 (2C), 149.75, 168.46 (2C); ESI–MS, m/z: 312.46 (2.36%), 313.21 (10.61%), 314.12 (M + , 27.68%), 269.28 (100.0%) Anal.Calcd for C_10_H_8_BrN_3_O_2_S (314.16) Elemental Analysis for C, 38.23; H, 2.57N, 13.38% found C, 38.23; H, 2.56; N, 13.39%.

#### 2,2′-bis(2-(furan-2-ylmethylene) hydrazinyl)-4,4′-bithiazole (10)

A mixture of thiosemicarbazone **1** (1.69 gm, 0.01mol), 1,4-dibromobutane-2,3-dione (4.87 gm, 0.02 mol) in absolute ethanol (20 ml, 99%) with trimethylamine (3drops) was refluxed for 10 h at (150 °C). (monitored by TLC), The precipitated solid formed on hot was filtered, washed with ethanol and dried. The crude product was recrystallized from ethanol to afford bis-thiazole derivative **10. **Black powder; yield 85 %; mp 300–305 °C; IR (KBr)*ν*/cm^−1^: 3269 (NH), 1620 (C=N); ^1^H NMR (DMSO-d6): *δ* (ppm): 6.62 (dd, 2H, J = 5 Hz, C4-furan), 6.811–6.819 (d, 2H, C3-furan), 6.98 (s, 2H, thiazole), 7.81 (s, 2H, C5-furan), 7.92 (s, 2H, =CH), 12.15 (s, 2H, NH); ^13^C NMR–(δC/ppm): 104.96 (2C), 112.55 (4C), 112.77 (2C), 132.13 (2C), 144.99 (2C), 149.77 (2C), 168.46 (2C); ESI-MS, m/z: 382.26 (M^+^, 56.09%), 372.76 (100.0%) Anal.Calcd for C_16_H_12_N_6_O_2_S_2_ (384.43). Elemental Analysis C, 49.99; H, 3.15; N, 21.86 % found C, 49.98; H, 3.14; N, 21.85 %.

#### 1,1′-(5-(furan-2-yl)-3-thioxo-1,2,4-triazolidine-1,4-diyl)bis(ethan-1-one) (11)

A mixture of thiosemicarbazone **1** (1.69 g, 0.01mol) and acetic anhydride (18 ml, 0.2 mol) was heated under reflux for 3 h at (120 °C), then cooled and poured onto ice water. The solid formed was filtered, dried and crystallized from ethanol to afford red powder **11**. Golden yellow solid; yield 90 %; mp 190–192 °C; IR (KBr)*ν*/cm^−1^: 3213 (NH), 1708, 1694 (C=O), 1288 (C=S); ^1^H NMR (DMSO-d6): *δ* (ppm): 2.04 (s, 3H, CH_3_), 2.14 (s, 3H, CH_3_), 6.31 (d, 1H, C3-furan), 6.38–6.39 (dd, 1H, J = 4.5 Hz, C4-furan), 6.90 (s, 1H, CH-triazole), 7.60 (d, 1H, C5-furan), 11.79 (s, 1H, NH); ESI-MS, m/z: 253.45 (M^+^, 50.40 %), 147.03 (100.0%) Anal.Calcd for: C_10_ H_11_N_3_O_3_S (253.05). Elemental Analysis C, 47.42; H, 4.38; N, 16.59% found C, 47.43; H, 4.37; N, 16.58%.

#### 2-(5-(2-(furan-2-ylmethylene) hydrazinyl)-1H-1,2,4-triazol-3-yl) acetonitrile (13)

A mixture of thiosemicarbazone **1** (1.69 gm, 0.01mol), 2-cyanoacetohydrazide (0.1gm, 0.01mol) in absolute ethanol (20 ml, 99%) was refluxed for 8 h at (130 °C). The reaction was poured onto ice. The precipitated solid was filtered, washed with ethanol and dried. The crude product was recrystallized from ethanol to afford compound **13**. Brown solid; yield 92 %; mp 90–92 °C; IR (KBr)*ν*/cm^−1^: 3367 (2NH), 2260 (CN), 1697 (C=N), ^1^H NMR (DMSO-d6): *δ* (ppm): 3.56 (s, 2H, CH_2_), 6.63–6.64 (dd, 1H, J = 4.8 Hz, C4-furan), 6.64–6.65 (d, 1H, C3-furan), 7.98 (d, 1H, C5-furan), 8.23 (s, 2H, =CH), 9.31 (s, 1H, NH), 11.45 (s, 1H, NH), ^13^C NMR-(δC/ppm): 18.81, 112.75, 116.47, 133.32, 135.26, 145.50, 149.55, 162.24, 178.08; ESI-MS, m/z: 216.23 (M+, 22.78%), 103.85 (100.0%); Anal.Calcd for C_9_H_8_N_6_O (216.20) Elemental Analysis C, 50.00; H, 3.73; N, 38.87% found 50.01; H, 3.74; N, 38.88%.

#### 3-(furan-2-yl)-1,5-dioxo-1,5-dihydrobenzo[e] [1,3] oxazepine-4 (3H)-carbothioamide (15)

Furan-2-carbaldehyde thiosemicarbazone **(1)** (1.69 gm, 0.01mol) and (1.48 gm, 0.01 mole) from phthalic anhydride in 20 mL from dry toluene (99%) was mixed, refluxed, for 12–14 h at (130 °C). Golden yellow solid; yield 95 %; m.p 98–100 °C; IR (KBr)*ν*/cm^−1^: 3413, 3219, 3138 (NH_2_, NH), 1689 (C=O), 1223 (C=S). ^1^H NMR (DMSO-d6): δ (ppm): 6.63–6.64 (dd, J = 5.2 Hz, 1H, C4- furan), 6.98–6.99 (dd, J=3.2 Hz, 1H, C3-furan), 7.58–7.62 (m, 1H, Ar-H), 7.64 (d, 1H, C5-furan), 7.67–7.70 (m, 1H, Ar-H), 7.82–7.83 (d, 1H, Ar-H), 7.98 (s, 1H, Ar-H), 8.24 (s, 1H, CH), 11.45 (s, 2H, NH_2_), 13.17 (s, 1H, NH); ^13^C NMR-(δC/ppm): 61.61, 112.80, 113.26, 128.88 (2C), 131.25 (2C), 132.97, 133.32, 145.44, 149.85, 169.15 (2C), 178.20; ESI-MS, m/z: 317.47 (M+, 26.15%), 149.13 (100.0%); Anal.Calcd for C_14_H_11_N_3_O_4_S (317.32) Elemental Analysis C_,_ 52.99; H, 3.49; N, 13.24% found C, 52.98; H, 3.48; N, 13.25%.

#### N-(3-formylquinolin-2-yl)-2-(furan-2-ylmethylene) hydrazine-1-carbothioamide (17)

To a solution of Furan-2-carbaldehyde thiosemicarbazone **(1)** (1.69gm, 0.01mol) in AcOH (20 ml) and 2-chloroquinoline-3-carbaldehyde (1.91 gm, 0.01mol) was added. The mixture was refluxed for 2–3 h at (130 °C). (monitored by TLC), The reaction was poured onto ice. The solid product was filtered off, washed with EtOH (95%) Black solid; yield 88%; mp 240–243 °C; IR (KBr)*ν*/cm^−1^: 3385 (NH), 1651 (C=O), 1287 (C=S) ^1^H NMR (DMSO-d6): δ (ppm): 7.23 -8.09 (m, 6H, H-furan and Ar–H), 8.04–8.29 (m, 4H, H-furan and Ar–H), 9.09 (s, 1H, CHO), 12.4 (s, H, NH), 14.17 (s, 1H, NH); ESI–MS, m/z: 328.53 (M+, 8.84%), 179.36 (100.0%) Anal.Calcd for C_16_ H_12_ N_4_SO_2_ (324.36). Elemental Analysis for C, 59.25; H, 3.73; N, 17.27% found C, 59.25; H, 3.72; N, 17.26%.

### Biological studies

#### Grape insects rearing

Samples of Grape Thrips (*Retithrips syriacus*) adults and Honey dew moth (*Cryptoblabes gnidiella* Miller) were collected from grape leaves and fruits, respectively of Kafr Al-Baramoun Research Farm, Horticultural Research Institute, Dakahlia, Egypt. Grape seedlings were used to rear grape thrips in cages (1.0 × 0.5 × 1.0 m) under laboratory conditions. After confirmed the definition of insects in the Taxonomy Department of the Plant Protection Research Institute. Male and female HM adult pairs were placed in plastic boxes (20 × 20 × 10 cm) when they emerged. These boxes contained grape fruits for oviposition as well as a piece of moistened cotton wool soaked in a 10% honey solution. A delicate hair brush was used every day to extract the eggs^13^. After the eggs hatching, larvae were daily provided with grape fruits.

#### Spodoptera frugiperda rearing

Fall armyworm, *Spodoptera frugiperda* larvae were collected from a corn field in Dakahliah governorate and be sure is free from insecticides. These larvae were reared on fresh leaves of castor oil in climatic control chamber (25 ± 1 °C, 65% RH) till pupation. Pupae were kept in PVC container containing sand under same condition until adult emergence. After that, moths were collected and held in glass mason jars (30 moths per jar) contains cotton soaked in sugar solution for feeding and pieces of zigzag shaped papers to provide dark arena for eggs oviposition. Egg mass were collected and held in plastic containers 200 ml under the same conditions until hatching. After three generations reared under laboratory conditions, we start the treatments^42^.

#### Bioassay activity for grape insects

The toxicity of the new synthetic compounds (**1, 5, 7, 9, 10, 11, 13, 15** and **17**) on grape thrips (*R. syriacus*) and HM (*C. gnidiella*) under laboratory conditions was assessed by spray method according to^43^ with some modification. Five concentrations were prepared (5–800 μg/mL, for both insects) by using tween 80, 0.01% DMSO and distilled water, each concentration was replicated 3 times. For thrips, ten healthy thrips nymphs were placed on a disc of grape leave (4 cm diameter/replicate). For each treatment one milliliter of test solution was used. Two milliliters of the test solution were sprayed on ten *C. gnidiella* 2^nd^ instar larvae after they had been placed in a Petri dish with some grape fruits. In the control treatment, both insects were treated with distilled water containing only Tween 80 and 0.01% DMSO. Mortality was counted daily.

#### Bioassay activity for* Spodoptera frugiperda*

Ten synthetic compounds (**1, 5, 7, 9, 10, 11, 13, 15** and **17**) were tested its toxicity against *S. frugiperda* using spray method technique under laboratory conditions. Five concentrations in each treatment were prepared (Three replicates/concentration). Thirty 2^rd^ instar larvae of *S. frugiperda* were used in each treatment (each larva in a jar contains a piece of castor leaf to avoid cannibalism) (10D × 5H cm). In each concentration two milliliters were used on larvae and a suitable piece of castor leaf. Only water, tween 80 and 0.01% DMSO were used in control^42^. In all Insects mortality was counted every day and corrected by Abbot^44^. The LC_50_ and LC_90_ values, together with their confidence limits and the regression lines’ slope, were supplied by Finney^45^. The toxicity index was also calculated using the Sun equation^46^.

#### Biochemical investigation of *S. frugiperda*

The most toxic compounds (**5, 7, 9** and **11**) were used at LC_50_ concentrations for measuring some enzymes activity of *S. frugiperda*. One millilitre of each compound was sprayed on twenty-five *S. frugiperda* 3^rd^ instar larvae. The live individuals were collected and weighed after 3 days of treatment, then frozen in a suitable tube. To assess the insect enzymes acid phosphatase (ACP)^47^, Aspartate transferase (AST), Alanine aminotransferase (ALT)^48^ alkaline phosphatase (ALP)^49^, and acetylcholine esterase (AchE)^50^, samples were transported to the Plant Protection Research Institute’s analysis unit.

#### Statistical analysis

MINITAB®software (version Minitab®21.4.1) was used to analyze the data. Probity analysis was used to calculate the LC_50_ and LC_90_ values for each treatment.

#### Quantum calculations

The computational studies were conducted using BIOVIA Materials Studio (20.1) software. The geometry optimization of (Products) was carried out utilizing the DMol^3^ module to implemented the density functional theory (DFT) calculations with appropriate cutoff parameters like Generalized Gradient Approximation (GGA) with Perdew–Burke–Ernzerhof (PBE) to ready Extracting the Highest Occupied Molecular Orbital (HOMO) and Lowest Unoccupied Molecular Orbital (LUMO).

#### Docking studies

The prepared compound optimized to be ready docking using Molecular Operating Environment (MOE) software which employed to investigate the biological interaction bond within the active site of the target protein to matching the experimental results conducted in this work, the calculation methodology based on triangle matcher and rigid receptors and scoring refinement methodology is carried by GBVI/WSA dG to search the best poses stable and the most acceptable root mean square deviation (RMSD) values.

## Conclusion

With the aim of finding new structural leads acting as agents, the current work set out to synthesize and assess the molecular modelling and DFT efficacy of a few novel furan derivatives. According to the biological experiment results, compounds highly toxic compounds (**5, 7, 9, 11** and** 15)** exhibited the strongest activity against type of insects towards *Retithrips syriacus, Cryptoblabes gnidiella*, and *Spodoptera frugiperda*. The lowest ΔE of **9** suggests high chemical reactivity, which has the ability to facilitate interactions with biological targets (e.g., insect enzymes) by active efficient electron donation/acceptance. The results of docking demonstrated strong binding affinities across tested compound **5, 7, 9** and **11** with S scores ranging from − 5.42 to − 7.33 kcal/mol all exceeding the energy cutoff. These findings matching with the biological results indicates that these off-target interactions trigger a rapid physiological response in insects, likely via stress-induced pathway, leading to hyperactivation and overproduction of AchE.

## Supplementary Information

Supplementary Information.

## Data Availability

Data is provided within supplementary information files.
