# Supplementary material for: Synthesis, cytotoxic screening and molecular docking, DFT calculation of novel furan-heterocyclic derivatives as insecticidal agents
Source: Sci Rep. 2025 Jun 27;15:20324. doi: 10.1038/s41598-025-06248-7 (PMC12205076; doi:10.1038/s41598-025-06248-7)
Supplement: Supplementary file 1 — Supplementary Information. [file 41598_2025_6248_MOESM1_ESM.docx]

**Synthesis, Cytotoxic Screening and Molecular Docking, DFT calculation of Novel furan-heterocyclic derivatives as Insecticidal agents**

Hager G. El-kasabi, ^a^ Margret M. Girges, ^a^ Samira A. Abd El-Salam,^b^ Ahmed.E.Suliman^c^, Ghada E. Abdel-Ghani^a*^

^a^ Department of Chemistry, Faculty of Science, Mansoura University, Mansoura, Egypt;

^b^ Plant Protection Research Institute, Agricultural Research Center, Giza-12618, Egypt;

^c^ Burg Al-arab Petroleum Company (Burapetco) 204 A ST 287 New Maadi, Cairo Egypt

*Corresponding author E-mail: [ghadaemadalden@mans.edu.eg](mailto:ghadaemadalden@mans.edu.eg)

**Data Availability**

Data will be made available on request from Corresponding author E-mail: [ghadaemadalden@mans.edu.eg](mailto:ghadaemadalden@mans.edu.eg)


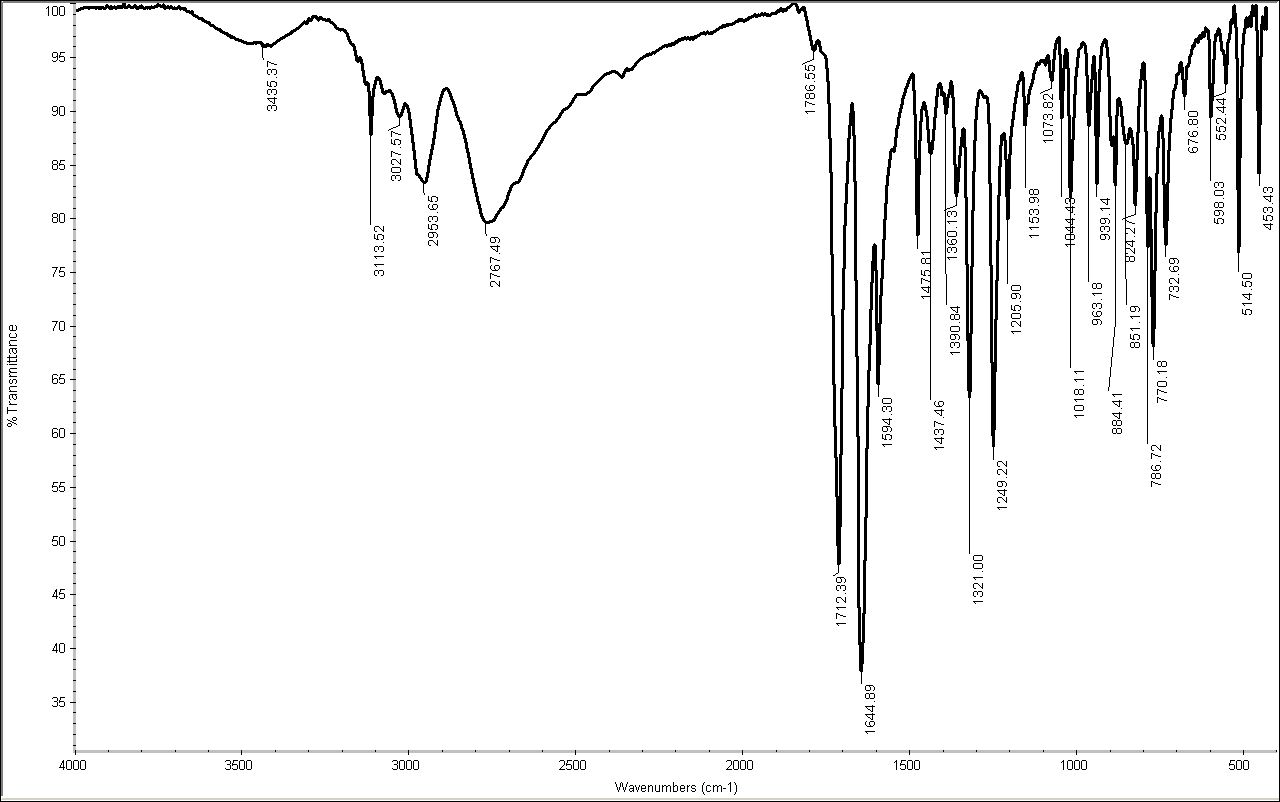

**Figure (S1):** IR Spectrum of compound **5**

**
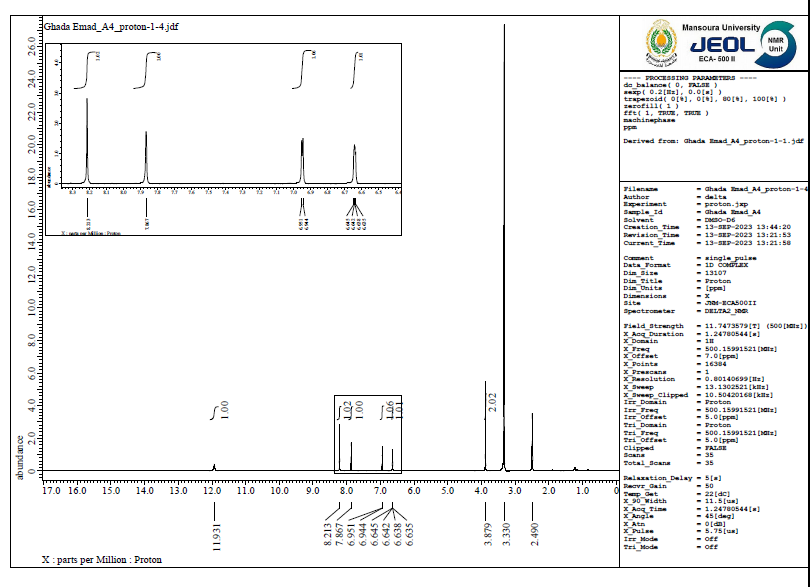
**

**Figure (S2):** ^1^HNMR (DMSO-*d*_6_, 500MHz) Spectrum of compound **5**

**
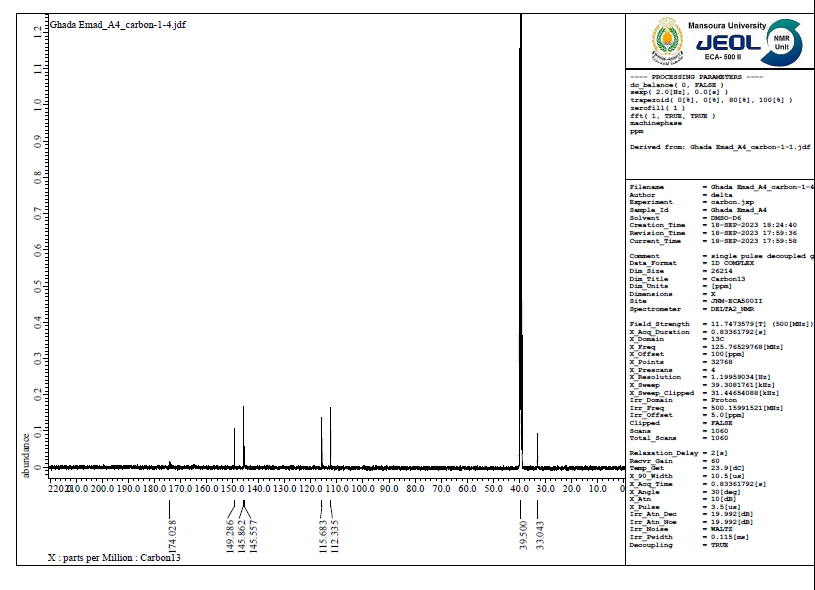
**

**Figure (S3):** ^13^CNMR (DMSO-*d*_6_, 125MHz) Spectrum of compound **5**

**Figure (S4):** Mass Spectrum of compound **5**

**
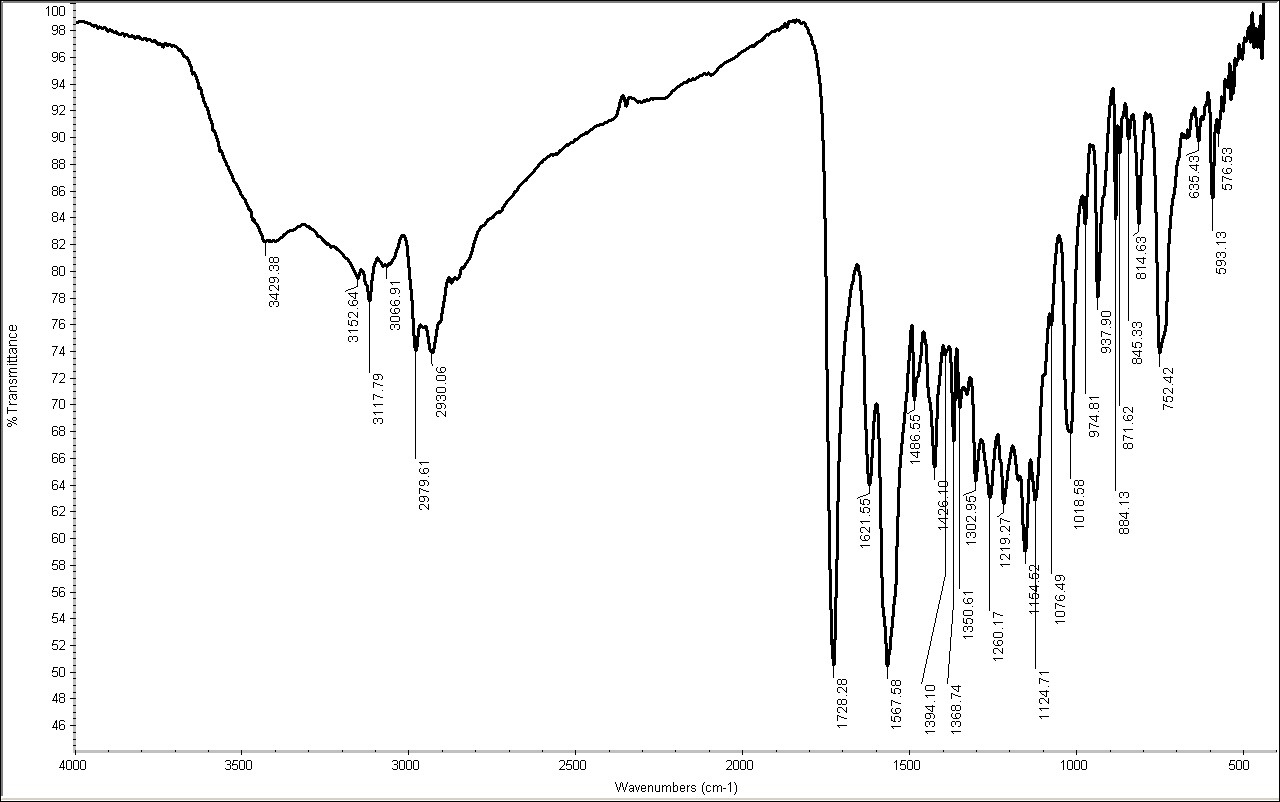
**

**Figure (S5):** IR Spectrum of compound **7**

**
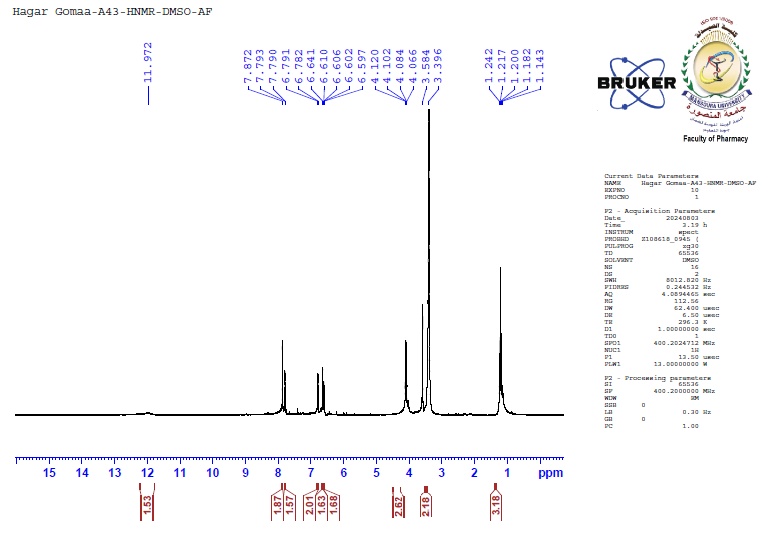
**

**Figure (S6):** ^1^HNMR (DMSO-*d*_6_, 400MHz) Spectrum of compound **7**

**
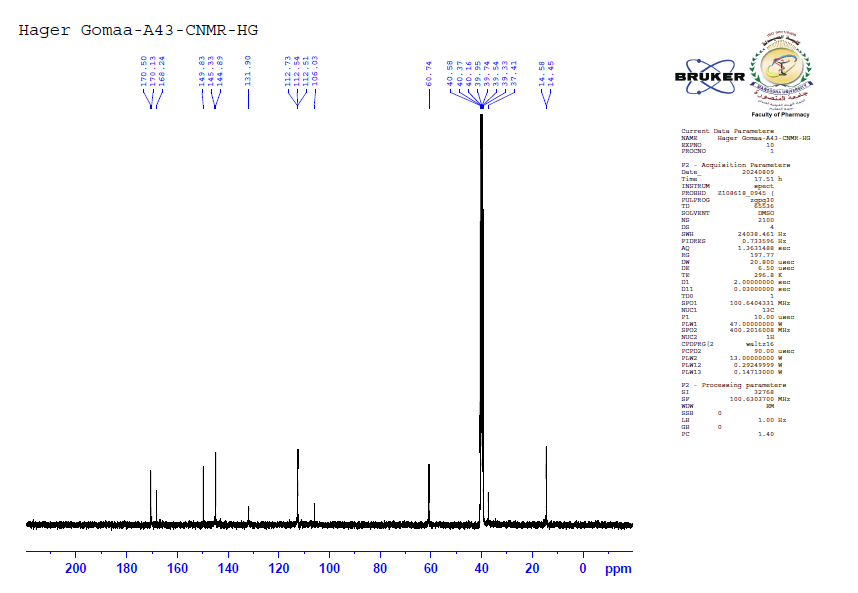
**

**Figure (S7):** ^13^CNMR (DMSO-*d*_6_, 100MHz) Spectrum of compound **7**

**Figure (S8):** Mass Spectrum of compound **7**

**
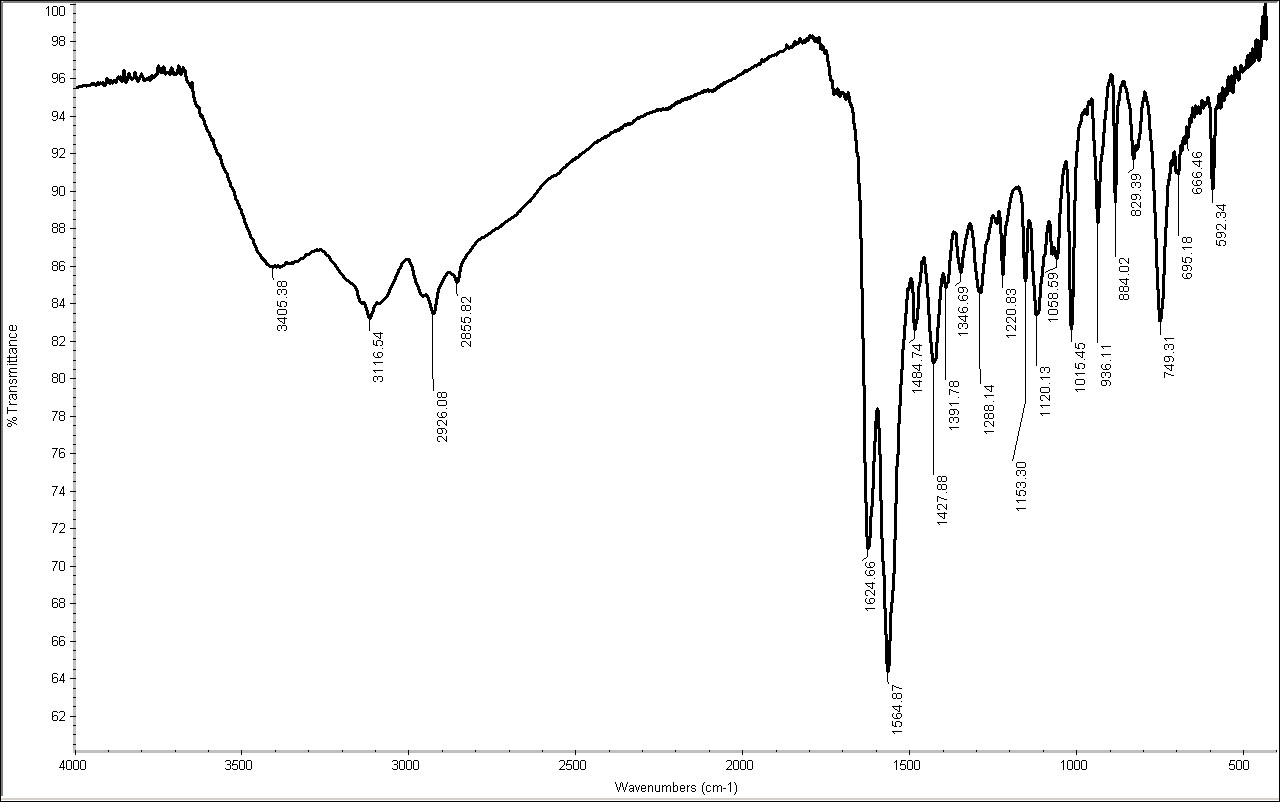
**

**Figure (S9):** IR Spectrum of compound **9**

**
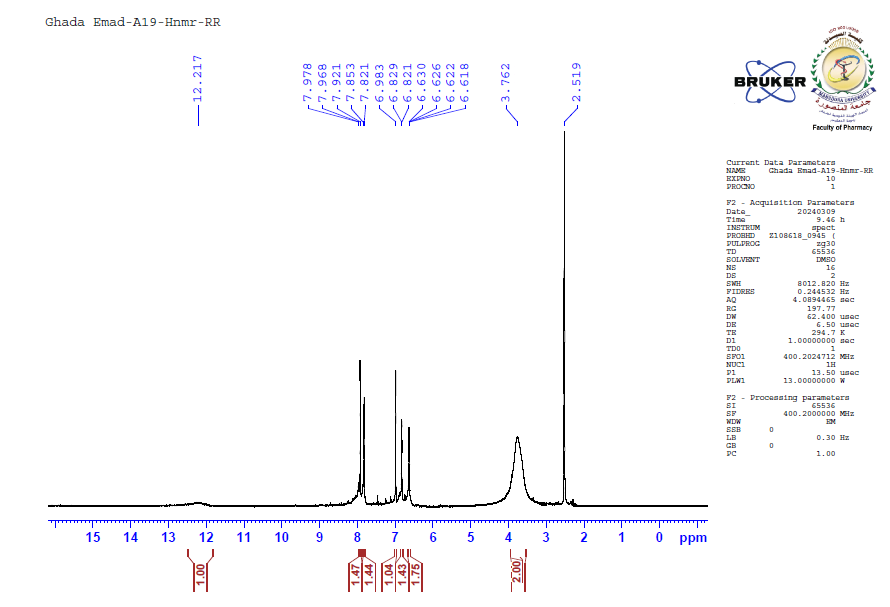
**

**Figure (S10):** ^1^HNMR (DMSO-*d*_6_, 400MHz) Spectrum of compound **9**

**
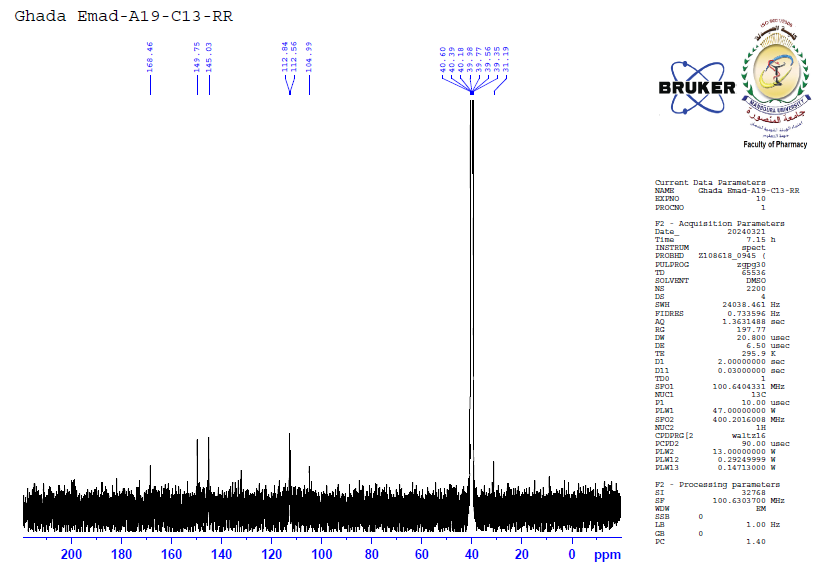
**

**Figure (S11):** ^13^CNMR (DMSO-*d*_6_, 100MHz) Spectrum of compound **9**

**Figure (S12):** Mass Spectrum of compound **9**

**
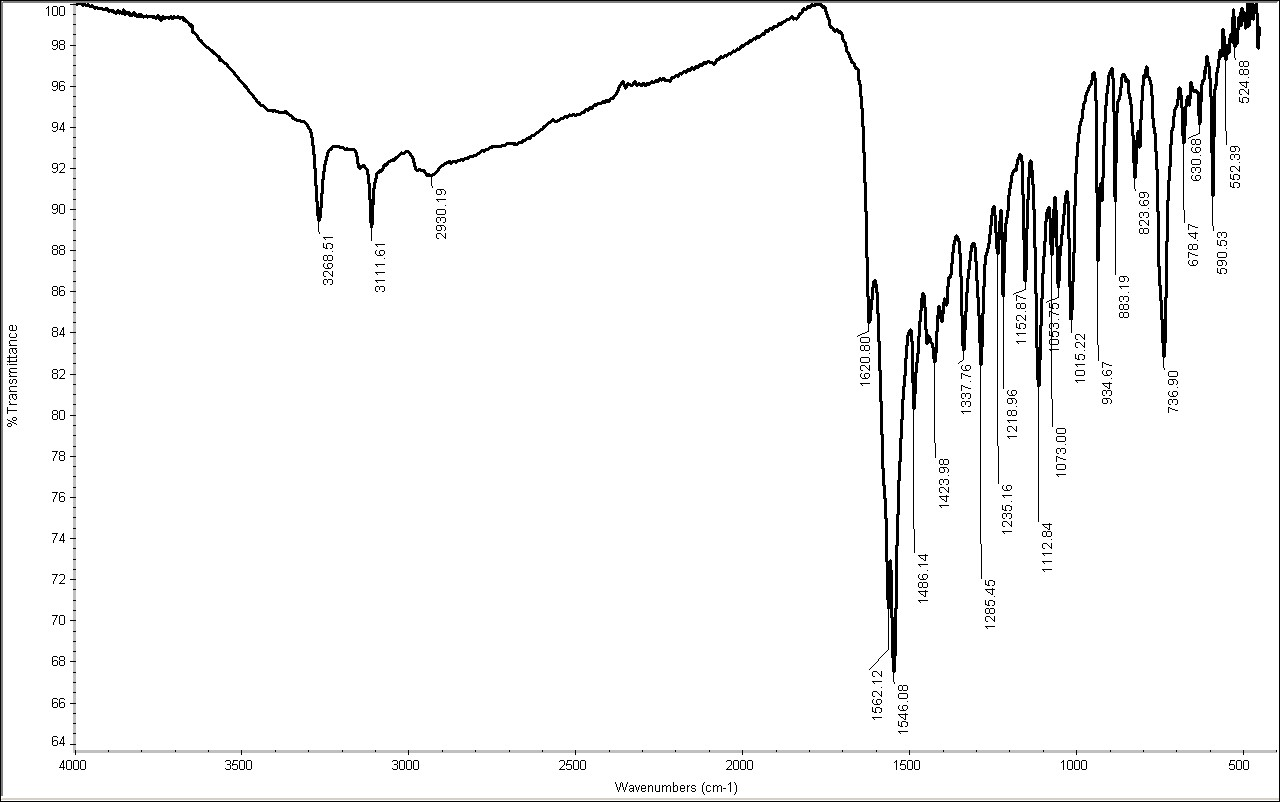
**

**Figure (S13):** IR Spectrum of compound **10**

**
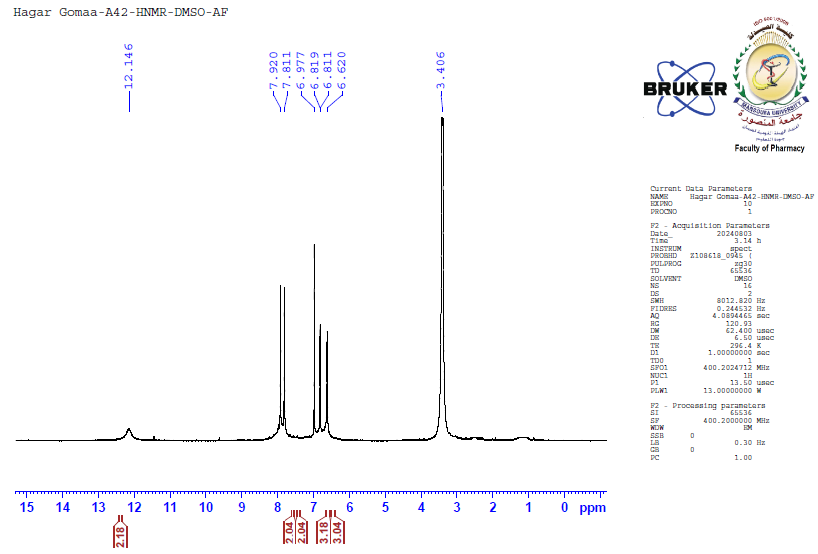
**

**Figure (S14):** ^1^HNMR (DMSO-*d*_6_, 400MHz) Spectrum of compound **10**

**
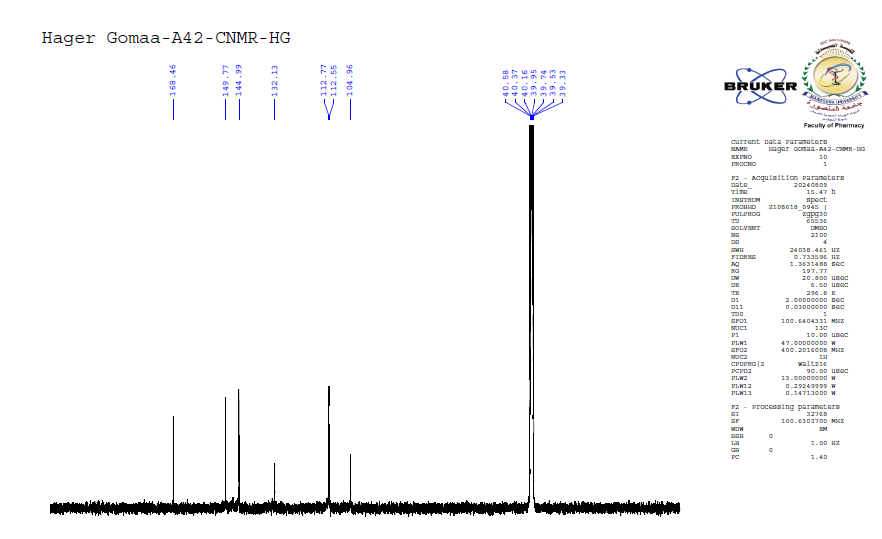
**

**Figure (S15):** ^13^CNMR (DMSO-*d*_6_, 100MHz) Spectrum of compound **10**

**Figure (S16):** Mass Spectrum of compound **10**

**
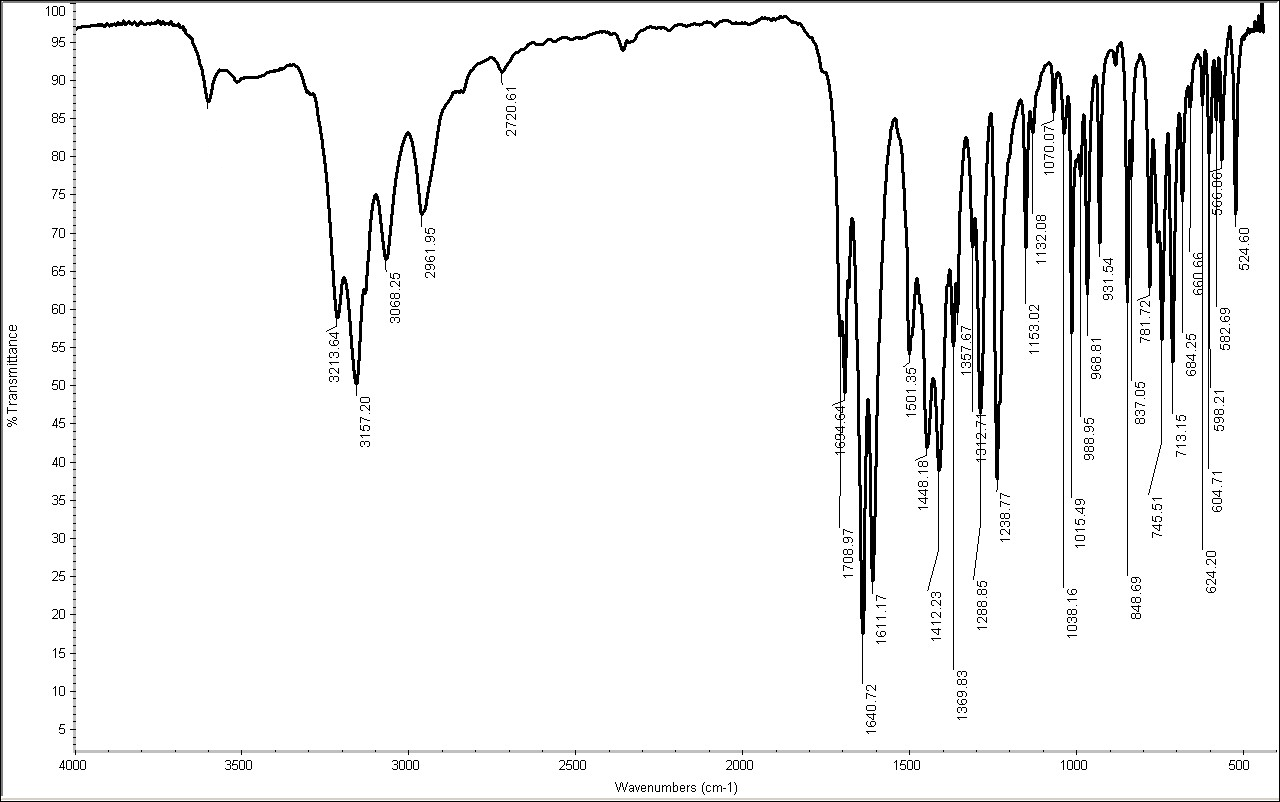
**

**Figure (S17):** IR Spectrum of compound **11**

**
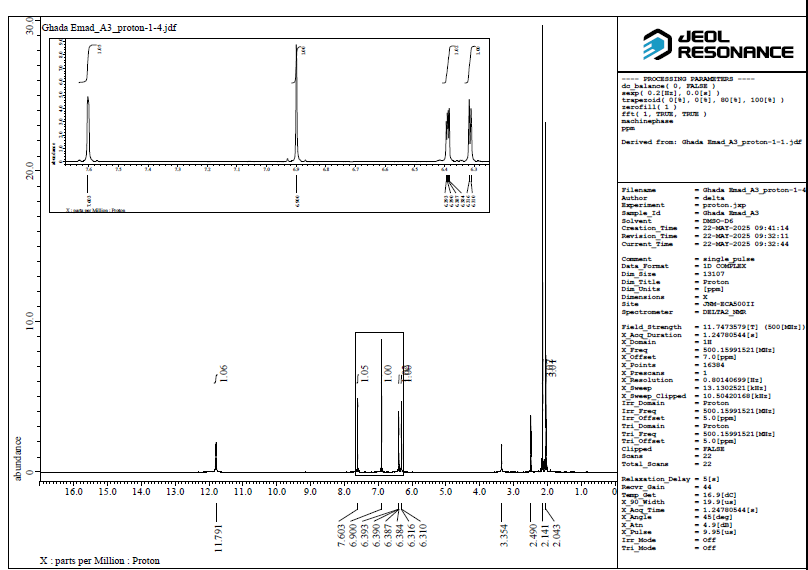
**

**Figure (S18):** ^1^HNMR (DMSO-*d*_6_, 500MHz) Spectrum of compound **11**

**
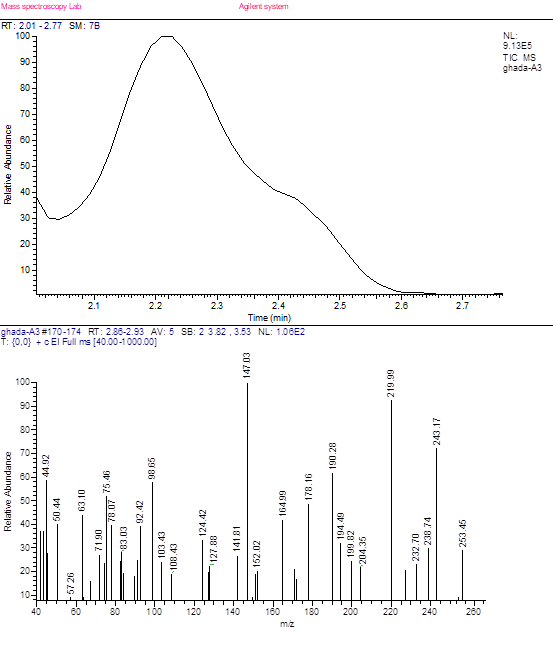
**

**Figure (S19):** Mass Spectrum of compound **11**

**
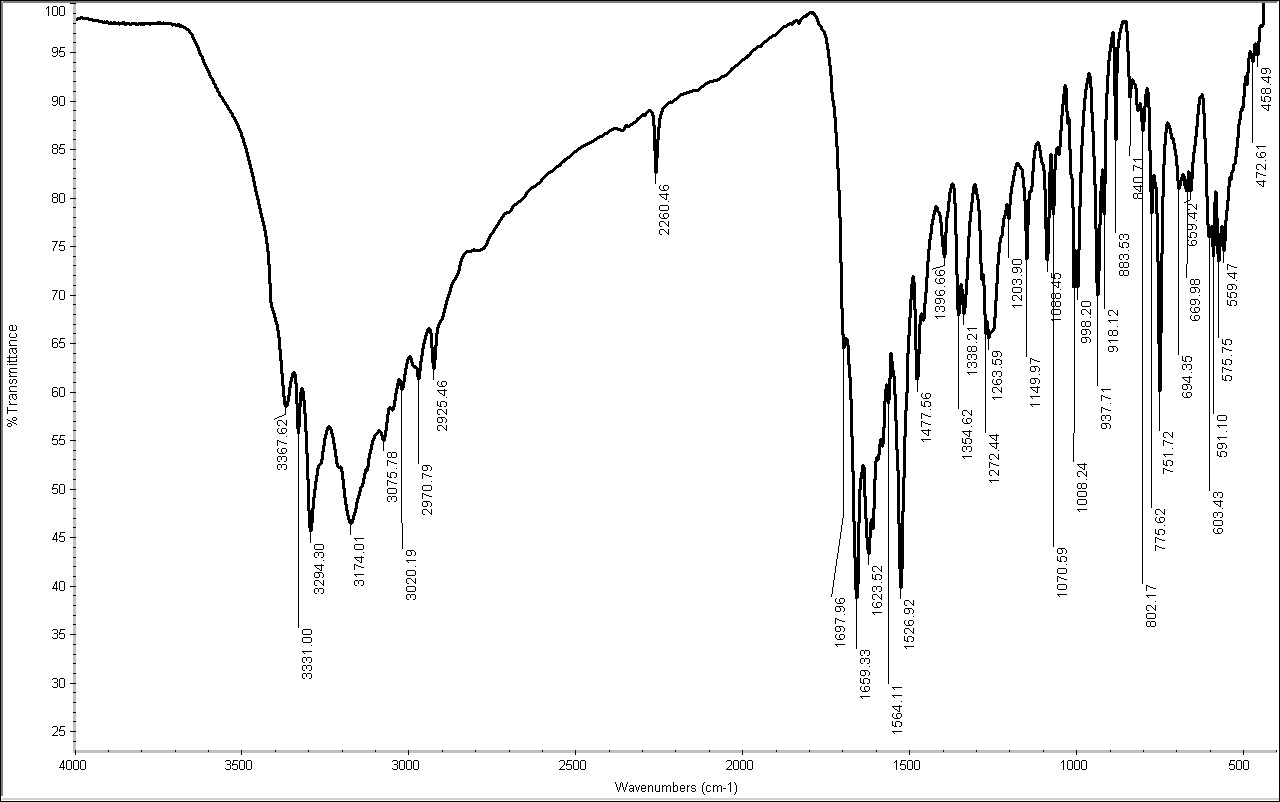
**

**Figure (S20):** IR Spectrum of compound **13**

**
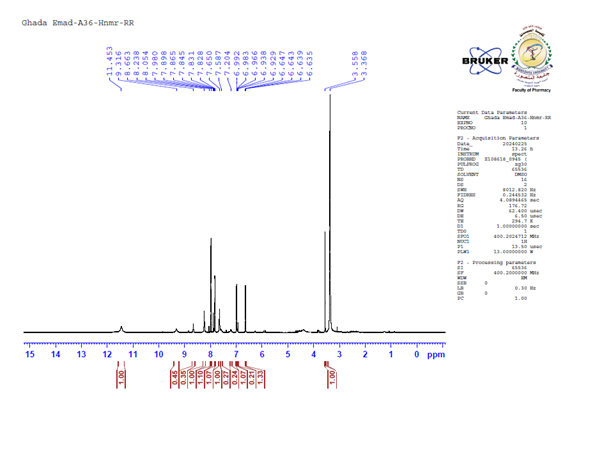
**

**Figure (S21):** ^1^HNMR (DMSO-*d*_6_, 400MHz) Spectrum of compound **13**

**
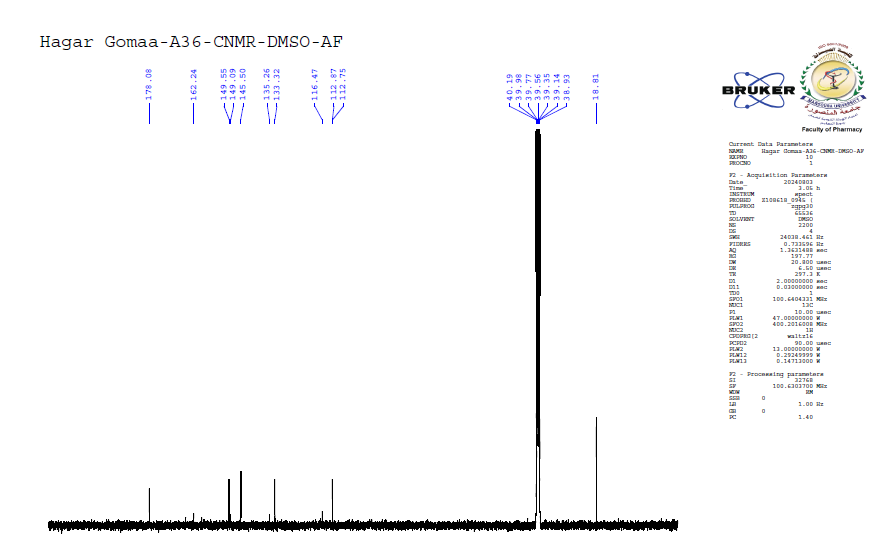
**

**Figure (S22):** ^13^CNMR (DMSO-*d*_6_, 100MHz) Spectrum of compound **13**

**Figure (S23):** Mass Spectrum of compound **13**

**
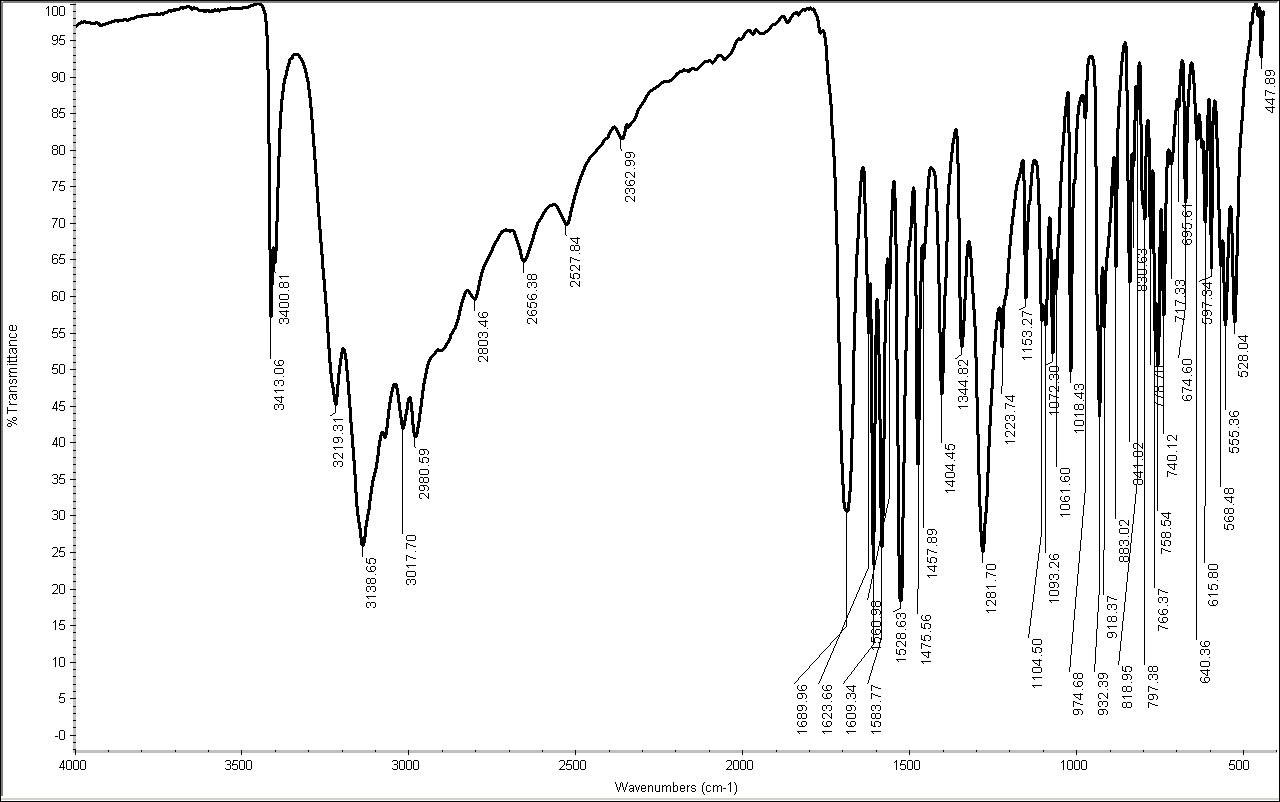
**

**Figure (S24):** IR Spectrum of compound **15**

**
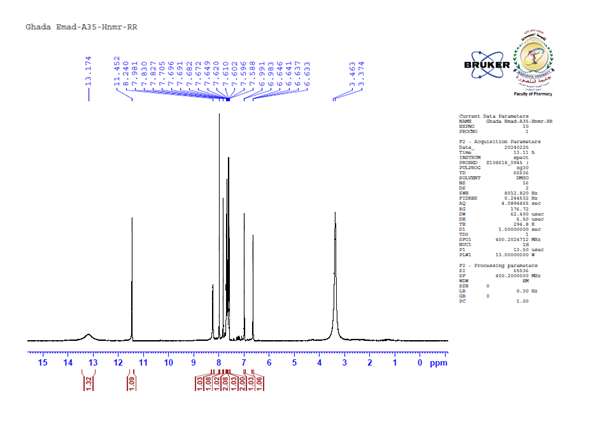
**

**Figure (S25):** ^1^HNMR (DMSO-*d*_6_, 400MHz) Spectrum of compound **15**

**
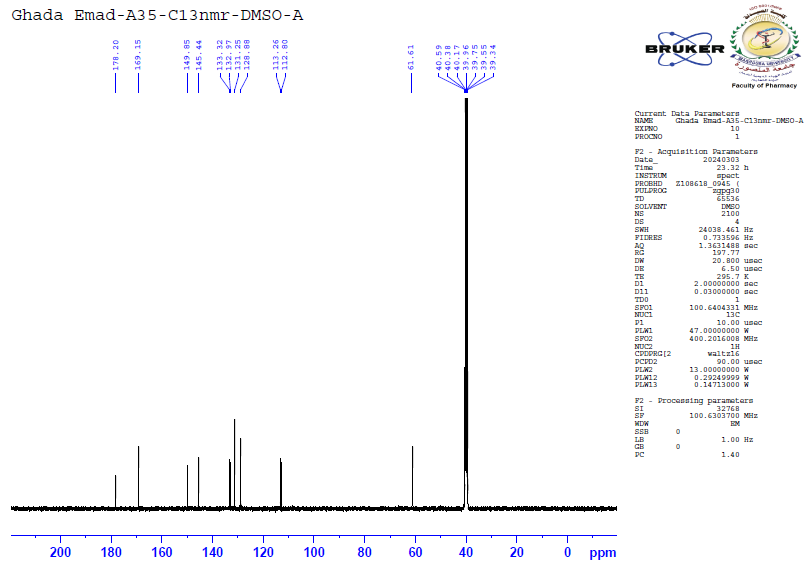
**

**Figure (S26):** ^13^CNMR (DMSO-*d*_6_, 100MHz) Spectrum of compound **15**

**Figure (S27):** Mass Spectrum of compound **15**

**
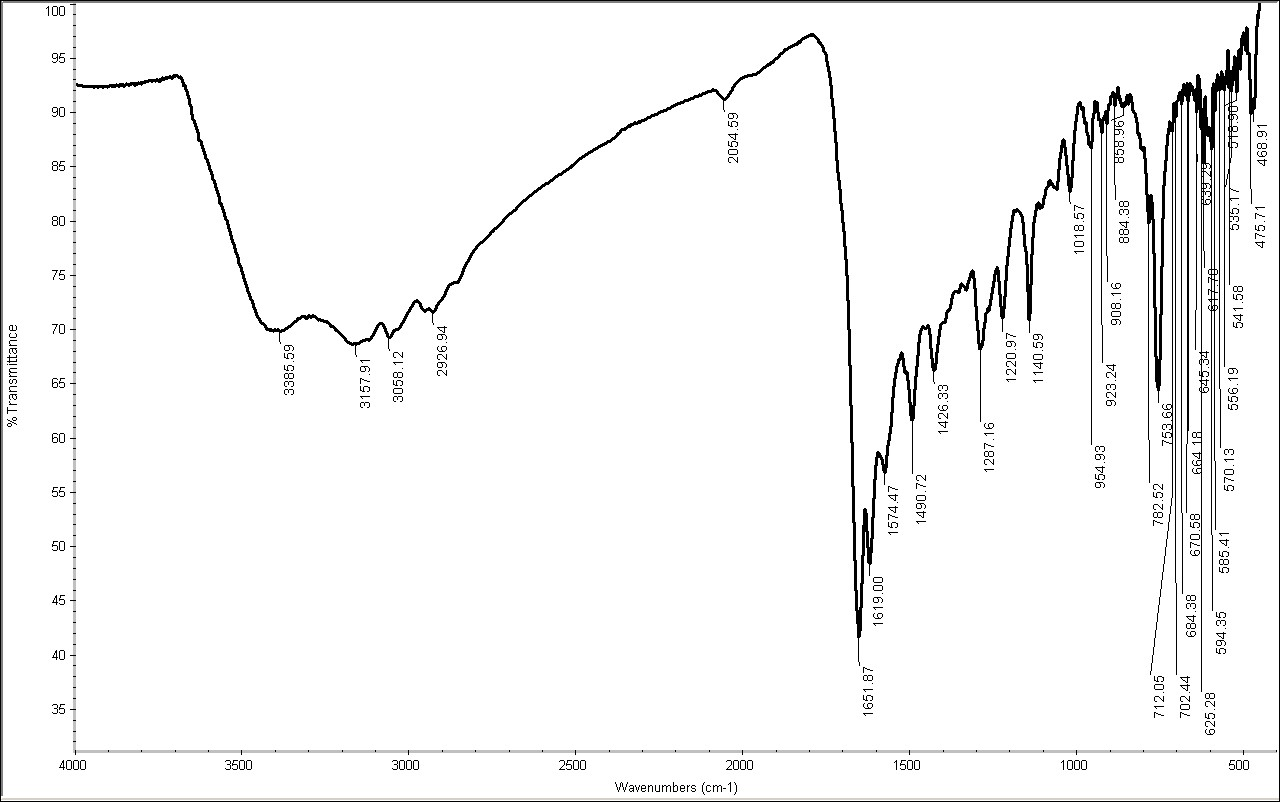
**

**Figure (S28):** IR Spectrum of compound **17**

**
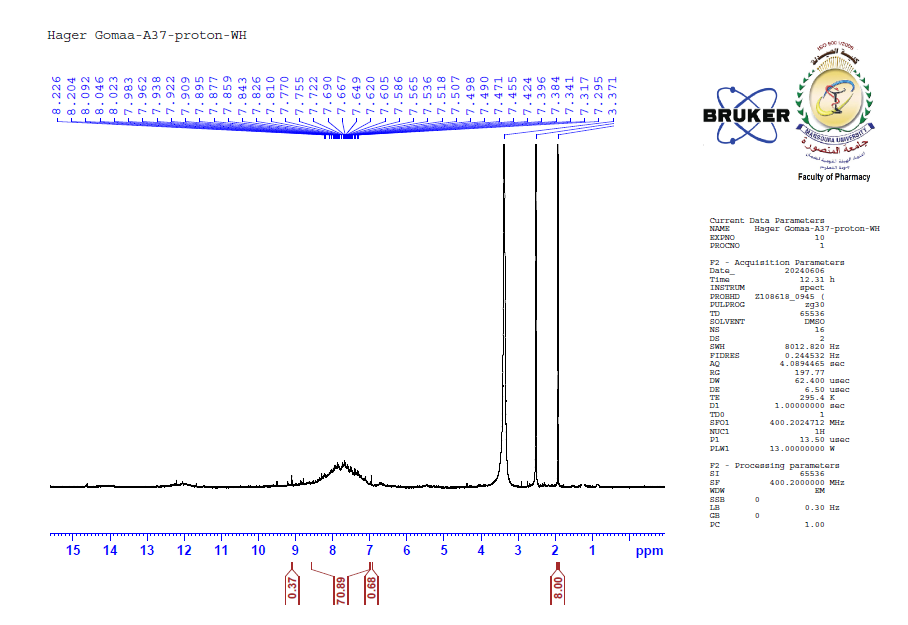
**

**Figure (S29):** ^1^HNMR (DMSO-*d*_6_, 400MHz) Spectrum of compound **17**

**Figure (S30):** Mass Spectrum of compound **17**
